# Hepatitis B surface antigen hijacks TANK-binding kinase 1 to suppress type I interferon and induce early autophagy

**DOI:** 10.1038/s41419-025-07605-0

**Published:** 2025-04-15

**Authors:** Chuanjin Luo, Caijiao Ma, Gang Xu, Chengbo Lu, June Ma, Yu Huang, Longyu Nie, Chen Yu, Yongfang Xia, Zhiqiang Liu, Ying Zhu, Shi Liu

**Affiliations:** 1 State Key Laboratory of Virology, Modern Virology Research Center, College of Life Sciences, Wuhan University, Wuhan, China; 2 Department of Clinical Laboratory, Maternal and Child Health Hospital of Hubei Province, Tongji Medical College, Huazhong University of Science and Technology, Wuhan, China

**Keywords:** Innate immunity, Immune evasion, Autophagy

## Abstract

There are close links between innate immunity and autophagy. However, the crosstalk between innate immunity and autophagy in host cells infected with hepatitis B virus (HBV) remains unclear. Here, we reported that HBsAg suppressed type I interferon production and induced the accumulation of autophagosomes. HBsAg boosted TANK-binding kinase 1 (TBK1) phosphorylation and depressed interferon regulatory factor 3 (IRF3) phosphorylation ex vivo and in vivo. Mechanistic studies showed that HBsAg interaction with the kinase domain (KD) of TBK1 augmented its dimerization but disrupted TBK1–IRF3 complexes. Using the TBK1 inhibitor, BX795, we discovered that HBsAg-enhanced TBK1 dimerization, promoting sequestosome-1 (p62) phosphorylation, was necessary for HBV-induced autophagy and HBV replication. Moreover, HBsAg blocked autophagosome–lysosome fusion by inhibiting the synaptosomal-associated protein 29 (SNAP29) promoter. Notably, liver tissues from HBsAg transgenic mice or chronic HBV patients revealed that IFNβ signaling was inhibited and incomplete autophagy was induced. These findings suggest a novel mechanism by which HBsAg targets TBK1 to inhibit type I interferon and induce early autophagy, possibly leading to persistent HBV infection.

**Figure:**
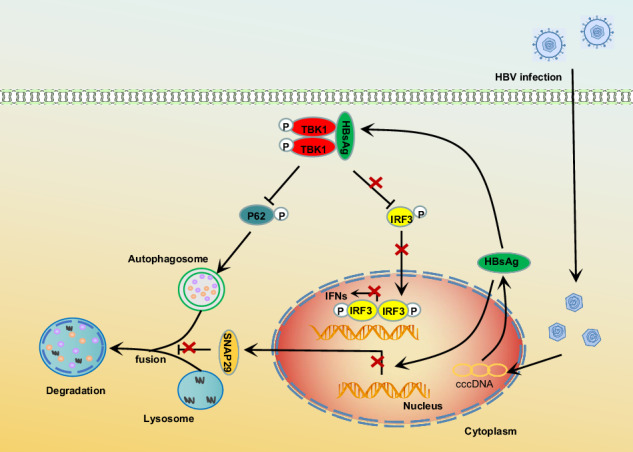
Molecular mechanisms of HBsAg suppression of the IFNβ signaling pathway and triggering of early autophagy. HBsAg targets the kinase domain of TBK1, thereby disrupting the TBK1–IRF3 complex and inhibiting type I interferon production. On the other hand, HBsAg enhances TBK1 dimerization and phosphorylation, which upregulates the phosphorylation of p62 to induce p62-mediated autophagy. Furthermore, HBV infection causes the accumulation of autophagosomes. This is achieved by HBsAg suppressing the SNAP29 promoter activity, which blocks autophagosome–lysosome fusion.

Molecular mechanisms of HBsAg suppression of the IFNβ signaling pathway and triggering of early autophagy. HBsAg targets the kinase domain of TBK1, thereby disrupting the TBK1–IRF3 complex and inhibiting type I interferon production. On the other hand, HBsAg enhances TBK1 dimerization and phosphorylation, which upregulates the phosphorylation of p62 to induce p62-mediated autophagy. Furthermore, HBV infection causes the accumulation of autophagosomes. This is achieved by HBsAg suppressing the SNAP29 promoter activity, which blocks autophagosome–lysosome fusion.

## Introduction

Approximately 350 million people worldwide are chronically infected with the hepatitis B virus (HBV), which puts them at risk of developing liver cancer [1]. The HBV contains partially double-stranded DNA of 3.2 kb. This DNA encodes a DNA polymerase (HBp), the surface antigen (HBsAg), the core antigen (HBc), and the X protein (HBx) [2]. The HBV surface protein is essential for the life cycle of HBV. This protein assembles the viral envelope and attaches to the functional receptor, sodium taurocholate cotransporting polypeptide (NTCP), which mediates cell entry [3]. It also allows mature HBV particles and subviral particles to be secreted [4]. There are three types of HBsAg: L (large), M (medium), and S (small); LHBs proteins have PreS2 and PreS1 structural domains at their N-terminus; MHBs proteins have a PreS1 at their N-terminus; and SHBs proteins lack the PreS domain. The three HBsAg proteins differ in their N-terminus but share a common S domain [5].

The host’s first defense against microbial invasion is the innate immune response. Studies have shown that pattern recognition receptors (PRRs), including toll-like receptors (TLRs), retinoic acid-inducible gene I (RIG-I)-like receptors (RLRs), and cyclic GMP-AMP synthase (cGAS), recognize HBV [6–8]. Upon viral infection, PRRs recruit adapters such as the TIR domain-containing adapter molecule 1 (TRIF), mitochondrial antiviral signaling protein (MAVS), and stimulator of interferon response cGAMP interactor 1 (STING) to facilitate the assembly of TRAF3–TBK1–IRF3 complex to induce type I and type III interferons (IFNs) expression [9]. IFNs then induce the expression of interferon-stimulated genes (e.g., ISG15, ISG56) to exert antiviral activity [10, 11]. HBsAg is associated with HBV immune escape. Mutations in the second loop of the ‘a determinant of HBsAg are the most commonly detected and are responsible for vaccination and immune escape [12]. A recent study shows that HBV surface antigen particles deliver hepatocyte microRNA to regulate monocyte activity and promote persistent viral infection [13]. In addition, studies have shown that HBV employs an aggressive strategy with multiple viral proteins to evade the innate immune response, including HBe, HBp, and HBx [14].

There is a close link between innate immunity and autophagy. Some innate immune signaling proteins are involved in autophagy, such as STING-mediated endoplasmic reticulum (ER)-golgi autophagy and MAVS-mediated mitochondrial autophagy [15, 16]. Many viral infections trigger autophagy to evade innate immunity. HSV-1, IAV, SARS-CoV-2, and porcine reproductive and respiratory syndrome viruses exploit autophagy to weaken immune defenses. They promote the autophagic degradation of innate immunity adapter proteins to diminish the immune response and maintain persistent infection [17–20]. Although HBV infection of liver cells has been reported to induce autophagy [21, 22], it is unclear whether autophagy is utilized to evade innate immunity.

TANK-binding kinase 1 (TBK1) is a protein kinase tightly regulated by dimerization, ubiquitination, and phosphorylation [23–25]. It plays a crucial role in innate immunity and autophagy [26]. When a pathogen invades, TNF receptor-associated factor 3 (TRAF3) ubiquitinates TBK1 to activate it, and activated TBK1 phosphorylates interferon regulatory factor 3 (IRF3) to induce type I interferon expression or sequestosome-1 (p62) to induce autophagy [27, 28]. P62 is a selective cargo receptor for autophagy-degrading misfolded proteins. It accumulates when autophagic degradation is suppressed. A study reported that TRIM23 promotes the dimerization of TBK1 during viral infection, which in turn facilitates the phosphorylation of serine at site 403 of p62 to initiate autophagy [27]. We hypothesized that the interaction between HBV and TBK1 might be associated with autophagy. To confirm this, we examined the effects of TBK1 activity on HBV autophagy and replication by knocking out TBK1 or treating it with BX795, a TBK1 inhibitor [29]. Several studies have shown that HBV-induced autophagy is incomplete [30, 31]. Lin et al. found that overexpression of synaptosomal-associated protein 29 (SNAP29) leads to HBV degradation [32]. The late stage of autophagy leads to HBV degradation, but silencing of late autophagy favors viral replication [33, 34]. Hence, HBV may employ specific tactics to impede autophagic degradation, aiding replication.

This study identified HBsAg as an immune escape protein that suppresses type I interferon production in vitro by targeting TBK1. We also discovered that HBsAg stimulates the accumulation of autophagosomes through TBK1. Our results reveal a novel mechanism by which HBV infection regulates the host’s innate immune response and early autophagy, possibly contributing to persistent viral infection.

## Materials and methods

### Statement of ethics

Ten HBV-infected patients and ten controls were recruited from Tongji Hospital (Wuhan, China). All patients and controls were selected for three independent replicated experiments without age or gender differences. This study was approved by the Institutional Review Board of Wuhan University approval and was conducted following the principles of the Declaration of Helsinki. Informed consent was obtained from the volunteers. Detailed information is provided in Table S1.

### Mice

The HBsAg transgenic mice C57BL/6J-TgN (AlblHBV) 44Bri (namely HBs-Tg mice) were kindly provided by Professor Jieliang Chen (Fudan University), who obtained the mice from Jackson Laboratory (Bar Harbor, ME), which contain a sequence encoding the HBV large envelope polypeptide (subtype, ayw) was placed downstream of the mouse albumin promoter. Age- and sex-matched wild-type (WT) C57BL/6 mice were purchased from the Center for Animal Experiments of Wuhan University. Mice were randomly allocated to experimental groups, and the investigator was blinded to the group allocation during the experiment. WT and HBs-Tg mice were injected with poly (I:C) (5 mg/kg body weight, dissolved in 1 ml/kg body weight of PBS) or PBS (1 ml/kg body weight) into the tail vein, and then the mice were sacrificed 24 h later for the corresponding experiments. Three transgenic and control mice were used for each experiment, and three experiments were conducted independently. All animal experiments followed the NIH Guide for the Care and Use of Laboratory Animals. Mice were housed in the specific pathogen-free animal facility at Wuhan University. HBs-Tg mice were performed with the following primers, and bands (250 bp) were shown in transgenic but not WT mice:

forward primer: 5′-AACATGGAGAACATCACATC-3′;

reverse primer: 5′-AGCGATAACCAGGACAAGTT-3′.

### Cell culture and transfection

Human-hepatoma cell lines (Huh7, HepG2, HepG2-hNTCP, and HepAD38) were purchased from the China Center Type Culture Collection. Huh7 is sensitive to interferon signaling pathways. It was extensively used to study the functional regions of viral proteins and virus-induced changes in signaling pathways in this study. HepG2 cells efficiently support cccDNA formation and HBV replication [35]. It was mainly used to harvest HBV (HepAD38) and infect HBV (HepG2-hNTCP) in this study. All media were supplemented with 10% heat-inactivated fetal bovine serum (FBS; Gibco, 10099141C), 100 U/ml penicillin, and 100 mg/ml streptomycin in a humidified incubator with 5% CO_2_ maintained at 37 °C. HepAD38 cells were supplemented with 10% heat-inactivated FBS, 100 U/ml penicillin, 100 mg/ml streptomycin, and 1 mg/ml adriamycin (Dox) in a humidified incubator with 5% CO_2_ maintained at 37 °C. According to the manufacturer’s instructions, HEK293T cells were transfected with polyethyleneimine (Sigma, 9002-98-6). THP-1, Huh7, and HepG2 cells were transfected with Lipofectamine 2000 (Invitrogen, 11668030).

### Plasmids, antibodies, and reagents

Mutant TBK1 (TBK1-D1(1-309), TBK1-D2(310-386), TBK1-D3(310-729), TBK1^EE^, TBK1^HIF^, TBK1^EE+HIF^, TBK1^K30R+k401R^, TBK1^S172A^) were generated using a pair of primers or primers containing a point mutation, and the products were subsequently enzymatically attached to the corresponding expression vector. The *SNAP29* promoter region (−2000 to +200) was subcloned directly into the luciferase pLG3-basic vector, and the expression region was subcloned directly into PKH3-3×HA vectors. DsRed-HBs and DsRed-HBc were subcloned directly into the pDsRed-Monomer-N1 vector. All constructs were confirmed by DNA sequencing. Primers used for PCR are listed in Table S2. GFP-LC3, mCherry-GFP-LC3, GFP-TBK1, GFP-IRF3, 3HA-TBK1, 3HA-TRIF, 3HA-STING, 3HA-MAVS, 3HA-IRF3, 3HA-IRF3-5D, Myc-TBK1, Myc-TRAF3, Myc-IRF3, 3HA-LHBs (large), 3HA-MHBs (middle), 3HA-SHBs (small), 3HA-PreS, 3HA-HBe, 3HA-HBc, Flag-HBs, Flag-TBK1, and IFNβ promoter plasmids were previously cloned by our laboratory [36–39]. A plasmid carrying a 1.3-fold length of HBV genome (pHBV-1.3; genotype D) was obtained from Dr. Robert Schneider (Baylor College of Medicine, Houston, Texas) and is maintained in our laboratory [37]. pHBV1.3-ΔHBs was constructed on the pHBV1.3 plasmid with the three translation initiation codons ATG mutated to ACG in the S region, resulting in the deletion of all HBsAg expression but not affecting polymerase expression. pHBV1.3-ΔHBs was a gift from Professor Hongzheng Yuan (Fudan University).

Antibodies against Flag (M185-3L), HA (M180-3), and Myc (M192-3) were purchased from Medical & Biological Laboratories. Antibodies against TBK1 (A3458), p-TBK1 (AP1418), IRF3 (A19717), p-IRF3 (AP1412), p62 (A19700), LC3B (A19665), BiP (A4908), ATF4 (A0201), ATG7 (A19604), SNAP29 (A4290), and LAMP1 (A21194) were purchased from ABclonal. Antibodies against GAPDH (60004-1-Ig), GFP (66002-1-Ig), and Lamin A/C (10298-1-AP) were purchased from ProteinTech. Antibodies against p-p62 (YP1505) were purchased from ImmunoWay. Antibodies against HBsAg (bs-1557G) were purchased from Bioss. Anti-Goat IgG-Cy3 (abs20028), anti-Mouse IgG-CY3 (abs20142), anti-Mouse IgG-FITC (abs20012), and anti-Rabbit IgG-FITC (abs20023) were purchased from Absin. Anti-Mouse IgG-HRP (BF03001), anti-Rabbit IgG-HRP (BF03008), and anti-Goat IgG-HRP (BF03015) were purchased from Biodragon.

Lipopolysaccharide (LPS; L2880), EBSS (E7510), and poly (I:C) (31852-29-6) were purchased from Sigma Aldrich, and HT-DNA (D8050) and Bafilomycin A1 (BafA1; A8510) were purchased from Solarbio. HT-DNA and poly (I:C) were used at a final concentration of 10 μg/ml. LPS was used at a final concentration of 4 μg/ml. BX759 (A8222), Rapamycin (RAPA; A8167), Chloroquine (CQ; A8628), and 3-MA (3-methyladenine; A8353) were purchased from APExbio. RAPA, CQ, and BafA1 induced or inhibited autophagy at the indicated concentrations. RAPA-stimulated cells were subjected to fluorescence analysis after 12 h, and immunoblotting analysis after 24 h, and CQ- and BafA1-stimulated cells were subjected to the corresponding analyses after 24 h. The China Center provided the SeV and IAV/PR/8/34 (H1N1) strains for the Type Culture Collection. Recombinant VSV (VSV-GFP) and recombinant HSV carrying the GFP gene (HSV-GFP) were provided by Professor Mingzhou Chen of Wuhan University.

### Generation of knockout cell lines

As described previously, Huh7 *IFNAR1* knockout cell lines were generated via CRISPR-Cas9. The following sequences targeting the *IFNAR1* gene were used [39]:

oligonucleotide1, 5′-CACCGACCCTAGTGCTCGTCGCCG-3′;

oligonucleotide2, 5′-AAACCGGCGACGAGCACTAGGGTC-3′.

The following sequences generated a Huh7 *TBK1* knockout cell line:

oligonucleotide3, 5′-AAACGCCAAGATGCAGAGCACTTC-3′;

oligonucleotide4, 5′-CACCGAAGTGCTCTGCATCTTGGC-3′.

### RNA interference

Oligonucleotides targeting ATG7 sequences were cloned into the AgeI and BamHI sites of the pLKO.1 puro cloning vector with short hairpin RNA (shRNA) construct. The following target sequences were designed to target ATG7 mRNA:

1. 5’- GCCTGCTGAGGAGCTCTCCAT -3’;
2. 5’- GCTTTGGGATTTGACACATTT -3’;
3. 5’- CCCAGCTATTGGAACACTGTA -3’;

### RNA quantification

Total RNA was extracted with TRIzol reagent according to the manufacturer’s instructions (Invitrogen, 15596026). The cDNA was synthesized with TRUEscript H Minus M-MuLV Reverse Transcriptase (Aidlab Biotechnologies, PC1703). qRT-PCR assays were performed using the Bio-Rad CFX connect system with Universal SYBR Green Fast qPCR Mix (ABclonal, RK21203). The relative abundances of indicated mRNA were normalized by the level of GAPDH expression in each sample. Primers used for qPCR assays are listed in Table S3.

### Luciferase assays

Huh7 cells were seeded into 12-well plates and co-transfected with pRL-TK and IFNβ or *SNAP29* reporter and other indicated plasmids using matching transfection reagents described above. Luciferase activity was measured with the Dual-Luciferase Reporter Assay system according to the manufacturer’s instructions. Data were normalized for transfection efficiency by division of firefly luciferase activity by that of Renilla luciferase (Promega, E2920).

### Preparation of HBV from HepAD38 cells and infection with HepG2-hNTCP cells

HepAD38 cells were cultured for three days in DMEM/F-12 medium (Gibco, 11320033) supplemented with 10% heat-inactivated FBS, 100 U/ml penicillin, and 100 mg/ml streptomycin in an incubator at 37 °C. A 5% CO_2_ humidified incubator was used, and the supernatant of HepAD38 cells was concentrated 100-fold by ultracentrifugation as HBV inoculum. HBV storage titers (genome equivalent multiplicity) were determined by qPCR.

HepG2-hNTCP cells were cultured in primary hepatocyte maintenance medium (PMM) for 6 h, then inoculated with 1000-fold genomic equivalents of HBV and incubated in PMM containing 4% PEG 8000 (Sigma -Aldrich, 89610) at 37 °C for approximately 16 hours. The medium was changed every other day. Cells were harvested for study 7 days after infection.

### Immunoprecipitation (IP) and immunoblot

Cells or tissues were lysed with RIPA lysis buffer supplemented with a protease inhibitor cocktail (MedChemExpress, HY-K0012) for immunoblot. Bio-Rad Protein Assay measured the total protein concentration, and immunoblot analysis was performed with the indicated Abs. For IP, whole-cell extracts were collected and lysed in IP buffer containing 1.0% (*v*/*v*) Nonidet P-40; 50 mM Tris-HCl, pH 7.4; 50 mM EDTA; 150 mM NaCl; and a protease inhibitor cocktail. After centrifugation for 10 min at 12,000×*g* at 4 °C, the supernatants were collected and prewashed by adding protein A/G agarose (Pierce, 20421) before incubation for 2 h at 4 °C. Then IgG or indicated antibodies (2 μg) were added to supernatants for 4 h at 4 °C, captured by adding the protein A/G agarose (Pierce, 20421) before incubation for 4 h at 4 °C and the beads were washed five times with IP buffer containing 500 mM NaCl. Immunoprecipitates or whole-cell lysates were loaded and subjected to SDS-PAGE, transferred onto nitrocellulose membranes, and then blotted with indicated antibodies for immunoblot analysis.

### Native page

Huh7 cells were seeded into a 12-well plate, cultured overnight, treated as indicated, and then harvested with 50 μL of ice-cold lysis buffer (50 mM Tris-HCl, pH 7.5; 150 mM NaCl; and 0.5% NP-40 containing protease inhibitor cocktail). After centrifugation at 12,000×*g*, 4 °C for 10 min, the supernatant protein was quantified and loaded with 5× loading buffer (Servicebio, G2034-1ML), and 30 μg total protein was applied to 8.0% native gel (Servicebio, G2042-50T) for separation. After electrophoresis, proteins were transferred onto a nitrocellulose membrane for immunoblotting.

### Confocal microscopy

Huh7 or HepG2 cells were plated on collagen, incubated in 14-mm confocal dishes, transfected with indicated plasmids, and treated with indicated reagents. Cells were washed with 1× PBS three times, fixed with 4% paraformaldehyde, permeabilized with 0.3% Triton X-100, and blocked with PBS containing 3% BSA for 1 h at room temperature. Then, the cells were immunostained with the indicated primary Abs overnight at 4 °C, followed by incubation with the relevant dye-conjugated secondary Abs at 37 °C for 1 h. The nuclei were stained with 4’,6-diamidino-2-phenylindole for 3 min at 37 °C. The cells were imaged using a fluorescence microscope (Leica, Germany) with a 100× objective lens. To quantify the number of LC3-positive puncta and AO-red fluorescence intensity, 30 cells were recorded in randomly selected fields of view by fluorescence microscopy, and the average fluorescence values were analyzed by Image J. For protein co-localization, nine randomly selected fields of view were recorded under a fluorescence microscope, and protein co-localization was analyzed by ImageJ.

### Immunohistochemistry and immunofluorescence

Liver tissues were collected from patients or mice, as mentioned above. The expression of p-TBK1, p62, p-p62, p-IRF3, SNAP29, IRF3, and LC3 in liver tissue was visualized using immunohistochemical staining or immunofluorescence staining of thin liver tissue sections using the indicated antibodies. The analysis of the protein’s expression was conducted with ImageJ.

### Evaluation of autophagy flux

For the autophagy flux analysis using mCherry-GFP-LC3 fusion protein, 30 cells were randomly selected, and the percentage of yellow-positive puncta was equal to that of the ratio of yellow puncta to all LC3 puncta (yellow puncta and red-only puncta). Fluorescence values and number of puncta per cell were analyzed using ImageJ software. For the autophagy flux index measured with BafA1, the autophagy flux index was calculated as the ratio of the LC3-II expression level in the presence of BafA1 to the LC3-II expression level in the absence of BafA1. The LC3-II expression level was corrected by the expression level of each GAPDH using Image J. The autophagy flux index of the control group was corrected to 1.

### Transmission electron microscopy (TEM)

Huh7 cells were inoculated into 6-well plates for 12 h, then transfected with a plasmid expressing HBsAg and treated with or without inhibitors or with isolated mouse liver tissue. Cells or tissues were fixed with 2.5% glutaraldehyde overnight. It was then fixed with 1% aqueous OsO_4_ and 2% aqueous uranyl acetate. Uranyl acetate. After dehydration with ethanol and propylene oxide and embedded in polybed 812 resin (Polysciences, 025950-1), ultrathin (80 nm) sections were sectioned with 2% uranyl acetate and stained with 0.3% lead citrate and subjected to preparation for TEM (JEM-1400plus, Japan) observation. To quantify autophagosome-like vesicles, TEM was performed at a magnification of 15000 times in randomly selected fields of view, and the number of double-membrane autophagosomes was recorded for ten cells.

### Statistical analysis

All data were expressed as mean values ± standard deviation. Statistical analyses were performed using GraphPad Prism 8 (GraphPad, USA). The two-tailed Student’s *t*-test was used to determine significant differences. Differences were considered statistically significant at *P* < 0.05.

## Results

### HBsAg negatively regulates IFNβ through TBK1

To assess the role of HBsAg in the innate immune response, we stimulated or infected human monocytic leukemia (THP-1) cells with LPS, poly (I:C), HT-DNA, or with SeV, HSV-1. A trend of down-regulation of *INFB1* mRNA with HBs (unspecified; most of the experiments used LHBs) expression gradient was found (Fig. S1A, B). Subsequently, we found that three types of HBsAg (LHBs, MHBs, and SHBs) decreased the SeV-triggered promoter activity and mRNA of *IFNB1* (Fig. S1C). TRIF, RIG-I, or cGAS and STING-induced *IFNB1* promoter activity and mRNA were also repressed by HBs (Fig. S1D), while the pHBV1.3-ΔHBs (HBsAg expression deletion) did the opposite (Fig. S1E). To further confirm HBs inhibition of IFNβ expression, WT and IFNβ receptor 1 (*IFNAR1*) knockout Huh7 cells were infected with recombinant HSV-GFP or VSV-GFP viruses. HBs increased the replication of the recombinant HSV-GFP or VSV-GFP viruses (Fig. S1F). However, these effects disappeared in *IFNAR1* knockout Huh7 cell lines (Fig. S1F). These data demonstrate that HBsAg negatively regulates IFNβ production.

**Fig. 1:**
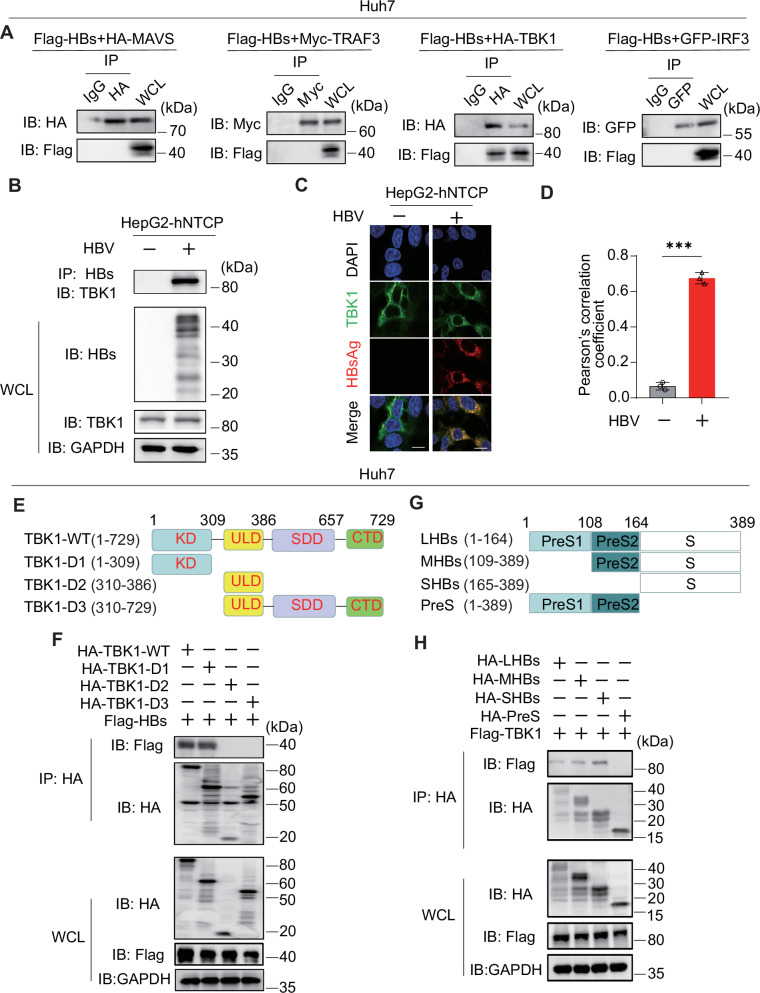
HBsAg interacts with TBK1. **A** IP and immunoblot analysis of Flag-HBs with HA-MAVS, Myc-TRAF3, GFP-IRF3, or HA-TBK1 in Huh7 cells. **B** IP and immunoblot analysis of endogenous TBK1 and HBsAg in HepG2-hNTCP cells infected with HBV. **C** Immunostaining analysis of endogenous TBK1 and HBsAg in HepG2-hNTCP cells infected with HBV. Scale bar: 10 μm. **D** Co-localization between HBsAg and TBK1 was analyzed by ImageJ. **E** Schematic of full-length TBK1 and its truncated mutants. **F** IP and immunoblot analysis of Flag-HBs with HA-TBK1 or its truncated mutants in Huh7 cells. **G** Schematic of large HBsAg (full-length HBsAg), middle HBsAg, small HBsAg, and PreS. **H** IP and immunoblot analysis of Flag-TBK1 with HA-LHBs, MHBs, SHBs, or PreS in Huh7 cells. All experiments were repeated at least three times with consistent results. Bar graphs show the means ± SD (*n* = 3 biological replicates). ^***^P < 0.001; using Student’s *t*-test.

To investigate how HBsAg inhibits IFNβ production, we examined the effects of HBsAg on IFNβ promoter activation mediated by various adapters. HBs strongly inhibited IFNβ reporter activation induced by TRIF, MAVS, STING, TRAF3, and TBK1 in a dose-dependent manner (Fig. S2A–E). However, IRF3-5D (an active phosphomimetic mutant of IRF3 in which the phosphomimetic aspartate replaced S396, S398, S402, S404, and S405 [40], Fig. S2F) was permissive to IFNβ reporter activation in the presence of HBs, suggesting that HBs suppress type I interferon by TBK1 or TBK1 downstream protein IRF3. Next, we investigated the interaction between HBs and TBK1. We co-transfected with Flag-HBs and HA-MAVS, Myc-TRAF3, HA-TBK1, or GFP-IRF3 into Huh7 cells. Flag-HBs coimmunoprecipitated with HA-TBK1 but not HA-MAVS, Myc-TRAF3, or GFP-IRF3 (Fig. 1A). The interaction between endogenous HBs and TBK1 was also observed in HBV-infected HepG2-hNTCP cells (Fig. 1B). Moreover, endogenous and exogenous TBK1 were co-localized with HBsAg (Figs. 1C, D and S2G, H). TBK1 has an N-terminal kinase domain (KD), a ubiquitin-like domain, an a-helical scaffold dimerization domain, and a C-terminal domain (CTD) [23] (Fig. 1E). To map which domain of TBK1 is required for the interaction with HBsAg, three kinds of TBK1 mutants were constructed (Fig. 1E). HBs was immunoprecipitated with TBK1-WT and TBK1-D1 but not with TBK1-D2 and TBK1-D3 (Fig. 1F). This result indicated that the KD was involved in the interaction (Fig. 1F). Three kinds of surface antigens, LHBs (large), MHBs (middle), and SHBs (small) (Fig. 1G), all inhibit type I interferon (Fig. S1C). Coimmunoprecipitation was performed to confirm whether they bind to TBK1. All the surface antigens interacted with TBK1 but not the PreS domain (164 amino acid sequence on the N-terminus of LHBs, Fig. 1G), suggesting that the S domain was required for the interaction (Fig. 1H). These findings indicate that HBsAg inhibits type I interferon by targeting and physically interacting with the KD of TBK1.

### HBsAg enhances TBK1 dimerization but inhibits IRF3 activation

**Fig. 2:**
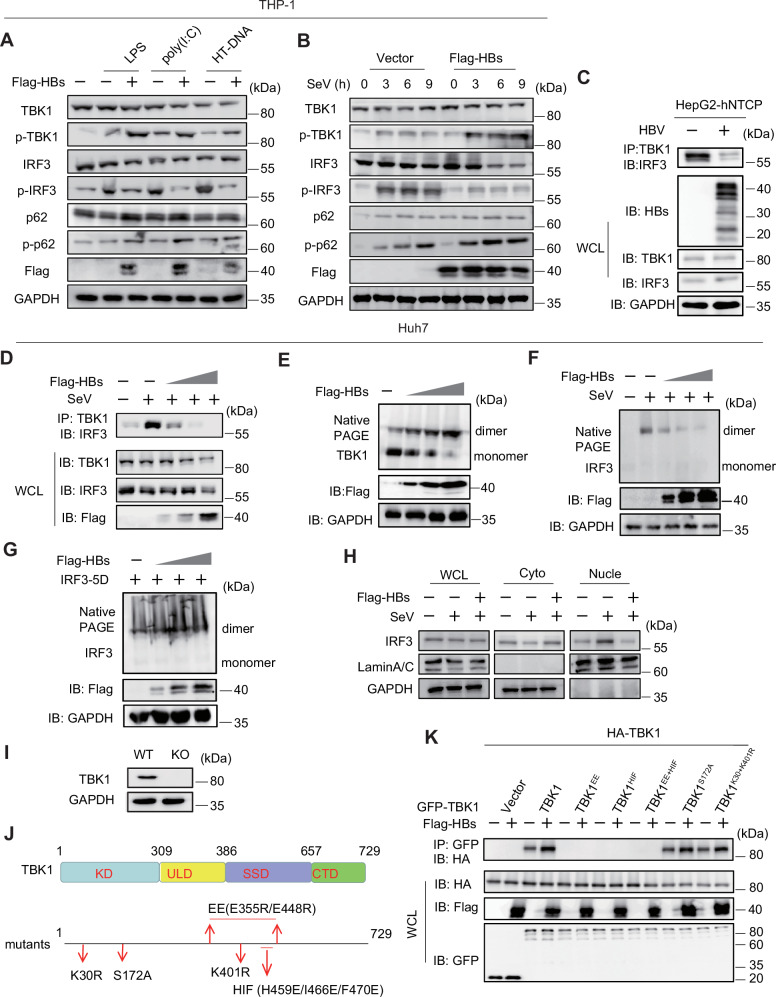
HBsAg enhances TBK1 dimerization but inhibits IRF3 activation. **A** Immunoblot analysis of phosphorylated (p-)TBK1, IRF3, and p62 in THP-1 cells transfected with a vector or Flag-HBs for 24 h and then stimulated with LPS, poly (I:C), or HT-DNA. **B** Immunoblot analysis of (p-) TBK1, IRF3, and p62 in THP-1 cells infected with SeV (MOI of 1) for the indicated times. **C** IP and immunoblot analysis of endogenous TBK1 and IRF3 in HepG2-hNTCP cells infected with HBV. **D** Huh7 cells were transfected with increasing amounts of a plasmid encoding Flag-HBs for 24 h and then infected with SeV (MOI of 1) for 8 h. IP and immunoblot analysis of endogenous TBK1 and IRF3. **E** Huh7 cells were transfected with increasing amounts of a plasmid encoding Flag-HBs for 36 h, followed by immunoblot analysis of TBK1 dimerization with native PAGE. **F** Huh7 cells were transfected with increasing amounts of a plasmid encoding Flag-HBs and then infected with SeV. Immunoblot analysis of IRF3 dimerization with native PAGE. **G** Huh7 cells were transfected with increasing amounts of a plasmid encoding Flag-HBs and IRF3-5D for 36 h. Immunoblot analysis of IRF3 dimerization with native PAGE. **H** Huh7 cells were transfected with increasing amounts of a plasmid encoding Flag-HBs and then infected with SeV, and immunoblot analysis of IRF3 in nuclear (Nucle) and cytoplasmic (Cyto) fractions. GAPDH served as a cytoplasmic control. LaminA/C served as a nuclear protein control. **I** Immunoblot analysis of TBK1 in WT and *TBK1* knockout Huh7 cells. **J** Schematic representation of TBK1 structural domains and critical amino acid sites. **K***TBK1* knockout Huh7 cells transfected with plasmids expressing HA-TBK1 (WT), GPF-TBK1 (WT), or its mutants (GFP-TBK1^EE^, TBK1^HIF^, TBK1^EE+HIF^, TBK1^S172A^, TBK1^K30R+K401R^), and along with or without Flag-HBs. IP analysis of TBK1 dimerization. All experiments were repeated at least three times with consistent results.

To investigate the effect of HBs on TBK1 and IRF3 phosphorylation, THP-1 cells were transfected with HBs-encoding plasmids and then stimulated or infected with LPS, poly (I:C), and HT-DNA (Fig. 2A) or with SeV for various times (Fig. 2B). Immunoblotting showed that HBs inhibited IRF3 phosphorylation but enhanced TBK1 phosphorylation. Early studies reported that TRAF3 activates TBK1 upon virus infection and that TBK1 and IRF3 then form a complex [9]. Coimmunoprecipitation was conducted to examine the potential inhibition of the TBK1–IRF3 interaction by HBs. HBs impeded the interaction between TBK1 and IRF3 but not TRAF3 (Fig. S3A–C). The interaction of endogenous TBK1 with IRF3 was blocked in HBV-infected HepG2-hNTCP (Fig. 2C). In agreement with these, HBs significantly disrupted SeV-induced TBK1–IRF3 interaction in a dose-dependent manner (Fig. 2D). Dimerized TBK1 phosphorylates IRF3 to dimerize and translocate it to the nucleus to induce IFN expression. Next, we performed IP and native PAGE immunoblots to explore the effects of HBs on TBK1 and IRF3. Dimerization of TBK1 was enhanced by HBs (Fig. 2E and S3D). However, dimerization of IRF3 (not IRF3-5D, Fig. 2F, G and S3E) and nuclear localization were significantly inhibited by HBs (Fig. 2H and S3F). TBK1 activation is a multi-step process: homodimerization, ubiquitination at the Lys30 and Lys401 sites, and trans-autophosphorylation at the Ser172. Homodimerization is a prerequisite for TBK1 activation, and mutants EE (double site in E355R/E448R) and HIF (triple site in H459E/I466E/F470E) abolish TBK1 dimerization [23, 24]. To further confirm that HBs promotes TBK1 dimerization, we constructed a *TBK1* knockout Huh7 cell line and TBK1 variants with these point mutations: Myc-TBK1^EE^ (E355R/E448R), Myc-TBK1^HIF^ (H459E/I466E/F470E), Myc-TBK1^HIF+EE^ (H459E/I466E/F470E and E355R/E448R combined), TBK1^S172A^, or TBK1^K30R+K401R^ (Fig. 2I, J). *TBK1* knockout Huh7 cells were transfected with these mutants and HA-TBK1 with or without Flag-HBs. TBK1, TBK1^S172A^, or TBK1^K30R+K401R^ were precipitated, except for TBK1^EE^, TBK1^HIF^, and TBK1^EE+HIF^ (Fig. 2K). These findings suggest that HBsAg enhances TBK1 dimerization but blocks the TBK1–IRF3 complex.

### TBK1 is required for HBsAg-induced accumulation of autophagosomes

**Fig. 3:**
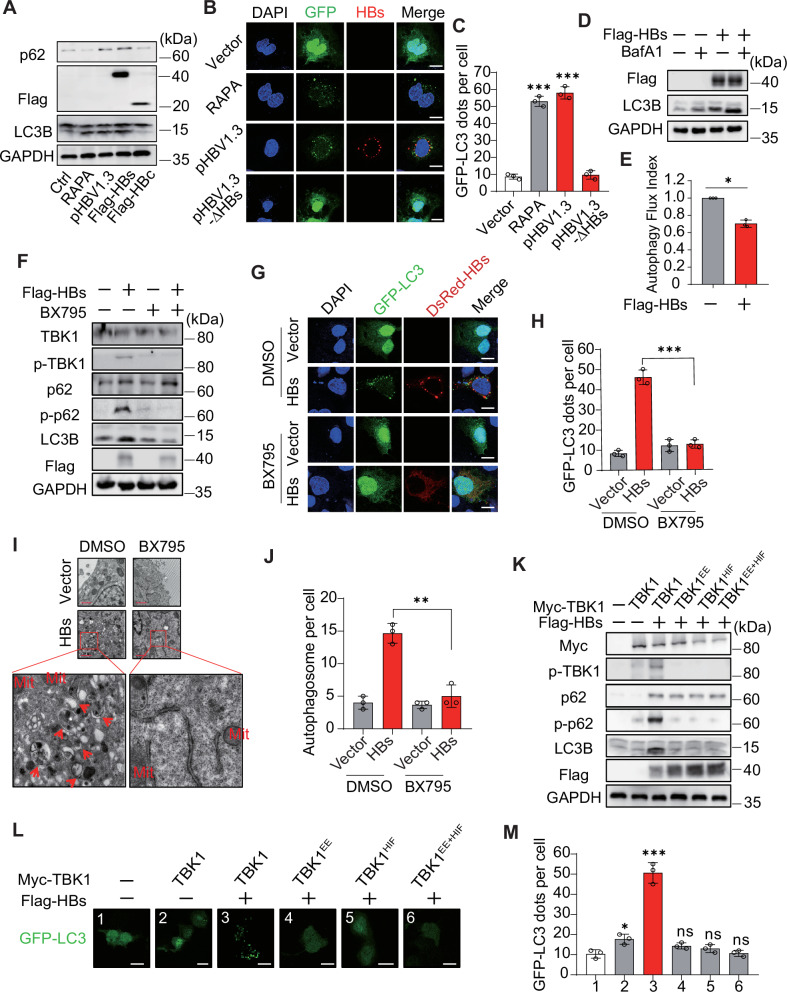
TBK1 is required for HBsAg-induced accumulation of autophagosomes. **A** HepG2 cells were transfected with pHBV1.3, Flag-HBs, or Flag-HBc plasmids for 36 h or treated with rapamycin (RAPA, 2.5 μM), followed by immunoblot analysis of p62 and LC3B proteins. **B** HepG2 cells were transfected with pHBV1.3 or pHBV1.3-ΔHBs plasmid along with GFP-LC3. LC3 puncta were examined by confocal microscopy. Scale bar: 10 μm. **C** The numbers of GFP-LC3 dots per cell in (**B**) were quantified. **D** Huh7 cells were transfected with Flag-HBs plasmid and treated with or without Bafilomycin A1 (BafA1, 100 nM). Immunoblot analysis of indicated proteins. **E** Autophagy flux indices were analyzed with and without transfection of HBs using Image J. **F** Huh7 cells were transfected with Flag-HBs plasmid and treated with or without BX759 (1 μM). Immunoblot analysis of indicated proteins. **G** Huh7 cells were transfected with GFP-LC3, and along with DsRed-HBs or vectors, the distribution of GFP-LC3 was imaged by confocal microscopy. Scale bar: 10 μm. **H** The numbers of GFP-LC3 dots per cell in (**G**) were quantified. **I** Cells were treated as in (**G**), and then TEM observe autophagosomes. Autophagosomes with double membranes are labeled with red arrows. Mit, mitochondria. Scale bar: 1 μm. **J** Quantitative analysis of the number of autophagosomes observed by TEM. **K***TBK1* knockout Huh7 cells were transfected with plasmids expressing Myc-TBK1, Myc-TBK1^HIF^, Myc-TBK1^EE^, Myc-TBK1^HIF+EE^, or an empty vector, along with Flag-HBs. Immunoblot analysis of the indicated protein. **L***TBK1* knockout Huh7 cells were transfected with plasmids encoding GFP-LC3, Flag-HBs, Myc-TBK1, Myc-TBK1^HIF^, Myc-TBK1^EE^, or Myc-TBK1^HIF+EE^. LC3 puncta were imaged by confocal microscopy. Scale bar: 10 μm. **M** The numbers of GFP-LC3 dots per cell in (**L**) were quantified. All experiments were repeated at least three times with consistent results. Bar graphs show the means ± SD (*n* = 3 biological replicates). ^*^P < 0.05; ^**^P < 0.01; ^***^P < 0.001; ns not significant; using Student’s *t*-test.

HBV-induced autophagy has been widely reported. MAP1LC3/LC3 conversion (LC3-I to LC3-II) and GFP-LC3 puncta are considered essential indicators of autophagy [41]. To determine whether HBs triggered autophagy, we transfected HepG2 cells with pHBV1.3, HA-HBs, or HA-HBc plasmids, and immunoblot showed that HBs but not HBc induced LC3 conversion (Fig. 3A). Further explorations revealed that all surface antigens (large, small, and middle) induced the LC3 conversion (Fig. S4A). Fluorescence microscopy also revealed that HBs induced GFP-LC3 puncta while pHBV1.3-ΔHBs (HBsAg expression deletion) did not (Fig. 3B, C and S4B, C). One study reported the partial presence of co-localization of LC3B and HBs, which was also observed in Fig. 3B and S4B [42]. Unlike complete autophagy induced by RAPA treatment, HBs or HBV upregulated p62 levels, although the trend in LC3-II was consistent (Fig. 3A and S4A), predicting that they may induce the initial phase of autophagy but impair the following autophagic degradation phase (late stage of autophagy). Next, to precisely capture the autophagy activity status, we constructed mCherry-GFP-LC3 fusion expression plasmids. During the early stage of autophagy, GFP and mCherry fluorescence are unaffected and show yellow fluorescence; during the late stage of autophagy, the acidic environment quenches GFP fluorescence to show mCherry fluorescence [41]. HBs and HBV produced yellow LC3 puncta, whereas RAPA produced several red puncta, suggesting that HBV impairs the autophagic degradation (Fig. S4D, E). Bafilomycin A1 (BafA1) can effectively inhibit autophagic lysosomal degradation and can be used to detect autophagic flux [43]. Figure 3D showed that HBs further elevated LC3-II levels after BafA1 treatment, suggesting that HBs induced the initial phase of autophagy. Furthermore, the autophagy flux index assessed by BafA1 was reduced after HBs transfection (Fig. 3E), suggesting that HBs inhibited the late stage of autophagy. Studies have shown that the accumulation of HBsAg causes ER stress [38, 44]. To investigate whether ER stress is the only pathway for HBs to induce autophagy, we performed immunoblot analysis of activating transcription factor 6 (ATF6) and binding-immunoglobulin protein (BiP), which increase expression after ER stress [38]. ATF6 and BiP were increased after transfection with HBV and HBs (Fig. S4F, G), indicating that HBs triggered ER stress. 4-phenylbutyric acid (4-PBA), an ER stress inhibitor, was used to counteract the unfolded protein response (UPR) triggered by HBs. Increased autophagosomes were observed by immunoblot, fluorescence microscopy, and TEM (Fig. S4H–L). These findings suggest that HBsAg may initiate autophagy through additional pathways.

Figure 2A, B showed that HBs increased TBK1 and p62 phosphorylation rather than IRF3. We hypothesized that HBs initiate autophagy through the TBK1-p62 axis during HBV infection. We transfected pHBV1.3 into Huh7 cells and found that LC3 conversion and p62 phosphorylation were upregulated (Fig. S4M). All kinds of HBs and HBV infections upregulated LC3 conversion and p62 phosphorylation (Fig. S4N, O). However, this phenomenon disappeared after treatment with BX795, a TBK1-targeting inhibitor [27] (Fig. 3F). Decreased autophagosomes were also observed by fluorescence and TEM following the BX795 treatment (Fig. 3G–J). These data suggest that HBs initiate autophagy through the TBK1-p62 axis. Figure 2K indicated that HBs increased TBK1 dimerization. Next, we explored the role of TBK1 dimerization in HBsAg-induced early phase of autophagy. We transfected *TBK1* knockout Huh7 cells with TBK1 (WT), Myc-TBK1^EE^, Myc-TBK1^HIF^, or Myc-TBK1^HIF+EE^. Compared to WT, *TBK1* knockout and these mutants abolished LC3-I/ΙΙ conversion, suggesting that TBK1 dimerization is required for HBsAg-induced early autophagy (Fig. 3K). Similar results were observed by fluorescence microscopy (Fig. 3L, M). These results suggest that HBs induce the early phase of autophagy through the TBK1-p62 axis but inhibit autophagic degradation, leading to the accumulation of autophagosomes.

### HBsAg blocks the autophagosome–lysosome fusion by silencing the SNAP29 promoter

**Fig. 4:**
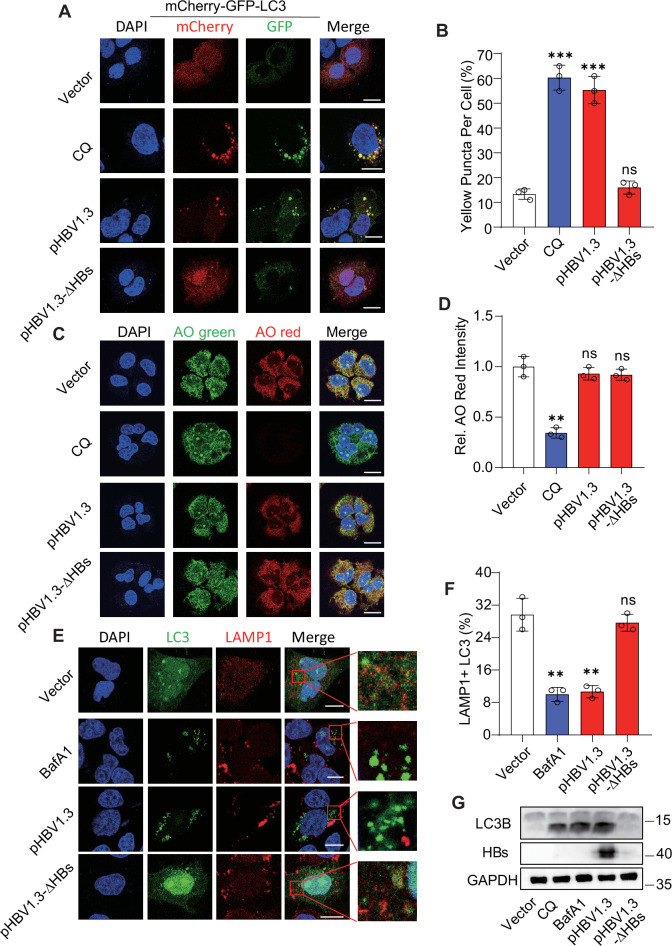
HBsAg blocks the autophagosome–lysosome fusion in HepG2 cells. **A** mCherry-GFP-LC3 expressing HepG2 cells were transfected with pHBV1.3, pHBV1.3-ΔHBs, or treated with CQ (10 μM). Cells were imaged by confocal microscopy. Scale bar: 10 μm. **B** Quantifying the percentage of yellow puncta/cell positive for both mCherry and GFP in cells. **C** HepG2 cells were transfected with pHBV1.3, pHBV1.3-ΔHBs plasmids or treated with CQ (10 μM). Cells were stained with AO and DAPI for 15 minutes and then detected by confocal microscopy. Scale bar: 20 μm. **D** Statistical analysis of relative AO-red intensity. **E** HepG2 cells were transfected with pHBV1.3, pHBV1.3-ΔHBs plasmids, or treated with BafA1 (100 nM). The co-localization of LC3 and LAMP1 was imaged. Scale bar: 10 μm. **F** Statistical analysis of co-localization between LC3 and LAMP1. The co-localization coefficient was represented as a percentage of puncta signals of LC3 that were positive for LAMP1. **G** HepG2 cells were transfected with pHBV1.3 or pHBV1.3-ΔHBs plasmids or stimulated with CQ or BafA1, and LC3B protein levels were analyzed by immunoblot. All experiments were repeated at least three times with consistent results. Bar graphs show the means ± SD (*n* = 3 biological replicates). ^**^P < 0.01; ^***^P < 0.001; ns not significant; using Student’s *t*-test.

The inhibition of autophagic degradation may be due to disruption of lysosomal acidification or blockade of autophagosome–lysosome fusion [41]. We performed an autophagic flux assay to explore how HBs inhibits autophagic degradation [41]. HBs induced the generation of GFP-LC3-II, but the GFP fragments remained unaffected [31] (Fig. S5A). Subsequently, the activity of autolysosomes was assessed. The acidic pH of lysosomes inhibits the GFP signal, while the mCherry signal remains unaffected [38]. CQ has been used as an autophagic degradation inhibitor that prevents the acidification of lysosomes [31]. We found that the quenching of the GFP signal was diminished with CQ stimulation or pHBV1.3 transfection but not pHBV1.3-ΔHBs (Fig. 4A, B). These results again suggest that HBs hindered autophagic degradation. Next, we checked lysosome acidification by assessing their acridine orange (AO) retention. AO emits green fluorescence in the cytosol but turns red when trapped in acidic lysosomes [38]. In contrast to CQ stimulation, the cytoplasmic AO-red did not decrease by pHBV1.3 (Fig. 4C, D). These results suggest that the acidic pH and the lysosomal enzyme degradation activity were not changed, and HBs may block autophagic degradation by preventing autophagosome–lysosome fusion. Subsequently, we found that transfection of pHBV1.3 yielded identical results as BafA1 (an autophagy inhibitor as a positive control [45]) treatment, with reduced co-localization of lysosome-associated membrane protein 1 (LAMP1) and LC3 (Fig. 4E, F), which were localized to autophagosomal and lysosomal membranes, respectively, and were used to indicate autophagosome–lysosome fusion [38]. Similar results were obtained by overexpressing HBs in Huh7 cells (Fig. S5B–G). LC3 conversion was assayed by immunoblot after CQ or BafA1 treatment, and an increased LC3-I/II conversion was found (Fig. 4G).

**Fig. 5:**
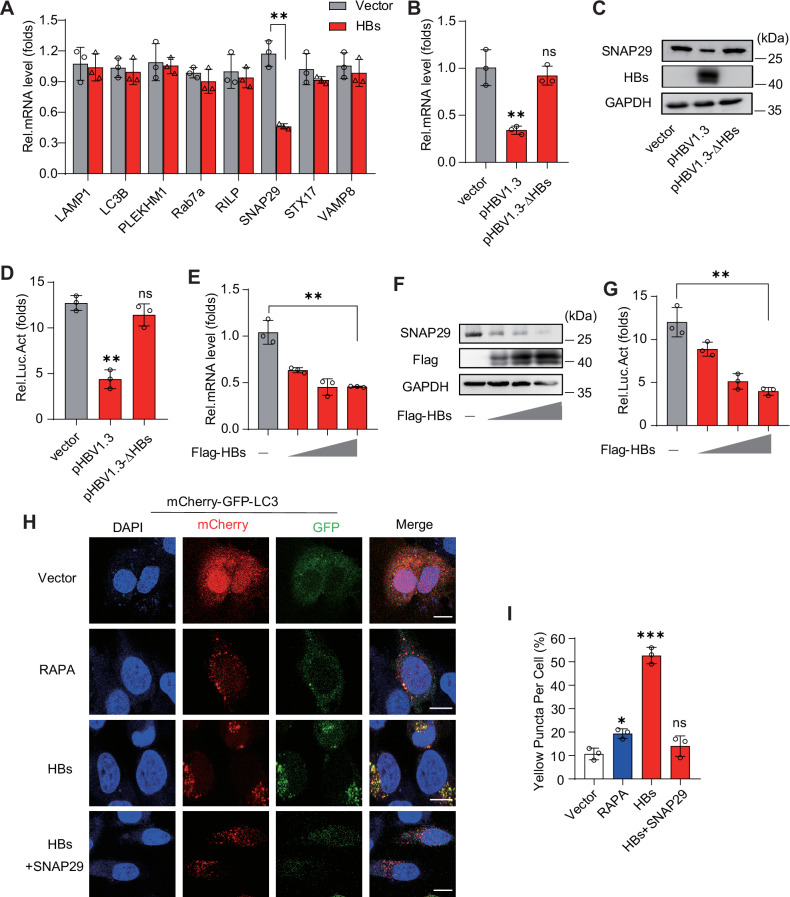
HBsAg suppresses the *SNAP29* promoter to induce incomplete autophagy. **A** Huh7 cells were transfected with a vector or plasmid expressing HBs for 48 h and then harvested for the indicated gene mRNA levels determination by qPCR. **B**, **C** Huh7 cells were transfected with pHBV1.3, pHBV1.3-ΔHBs, or vectors. *SNAP29* mRNA (**B**) and protein (**C**) levels were detected. **D** Huh7 cells were transfected with pHBV1.3, pHBV1.3-ΔHBs or vector, a *SNAP29* promoter, and a Renilla-TK reporter. *SNAP29* promoter activity was detected. **E**, **F** HepG2 cells were transfected with increasing amounts of a plasmid encoding Flag-HBs for 24 h. *SNAP29* mRNA (**E**) and protein (**F**) levels were detected. **G** HepG2 cells were transfected with increasing amounts of a plasmid encoding Flag-HBs, a SNAP29 promoter, and a Renilla-TK reporter. *SNAP29* promoter activity was detected. **H** HepG2 cells expressing mCherry-GFP-LC3 plasmid were transfected with HBs and with or without SNAP29 encoding plasmids or treated with RAPA (2.5 μM). Cells were imaged by confocal microscopy. Scale bar: 10 μm. **I** Quantifying the percentage of yellow puncta/cell positive for both mCherry and GFP in cells. All experiments were repeated at least three times with consistent results. Bar graphs show the means ± SD (*n* = 3 biological replicates). ^*^P < 0.05; ^**^P < 0.01; ^***^P < 0.001; ns not significant; using Student’s *t*-test.

To explore how autophagosome–lysosome fusion was hindered in hepatoma cells, HBs plasmids were transfected into Huh7 cells. Genes that determine autophagosome–lysosome fusion (*LAMP1*, *LC3B*, *PLEKHM1*, *Rab7a*, *RILP*, *SNAP29*, *STX17,* and *VAMP8*) were measured using qRT-PCR. *SNAP29* mRNA was significantly downregulated, but others were unaffected (Fig. 5A). In addition, pHBV1.3 validated that HBs decreased *SNAP29* mRNA and protein levels (Fig. 5B, C) but not pHBV1.3-ΔHBs. To delve deeper into the impact of HBs on *SNAP29* expression through transcription, we cloned and constructed the promoter region (−2000 to +200) of the human *SNAP29* gene into the luciferase pLG3-basic vector. The *SNAP29* promoter activation was also inhibited by HBs (Fig. 5D). A gradient transfection of HBs was conducted in HepG2 cells. Similar phenomena were found at the mRNA, protein, and promoter levels (Fig. 5E–G). To confirm that HBs inhibited autophagosome–lysosome fusion via SNAP29, SNAP29-encoding plasmids were transfected into HepG2 cells. We found that SNAP29 expression was identical to RAPA treatment, significantly attenuated GFP signaling accumulated by HBs, and mCherry signaling was unaffected (Fig. 5H, I). These findings suggest that HBs silence the *SNAP29* promoter to block autophagosome–lysosome fusion.

### HBV replication relies on TBK1 activity

**Fig. 6:**
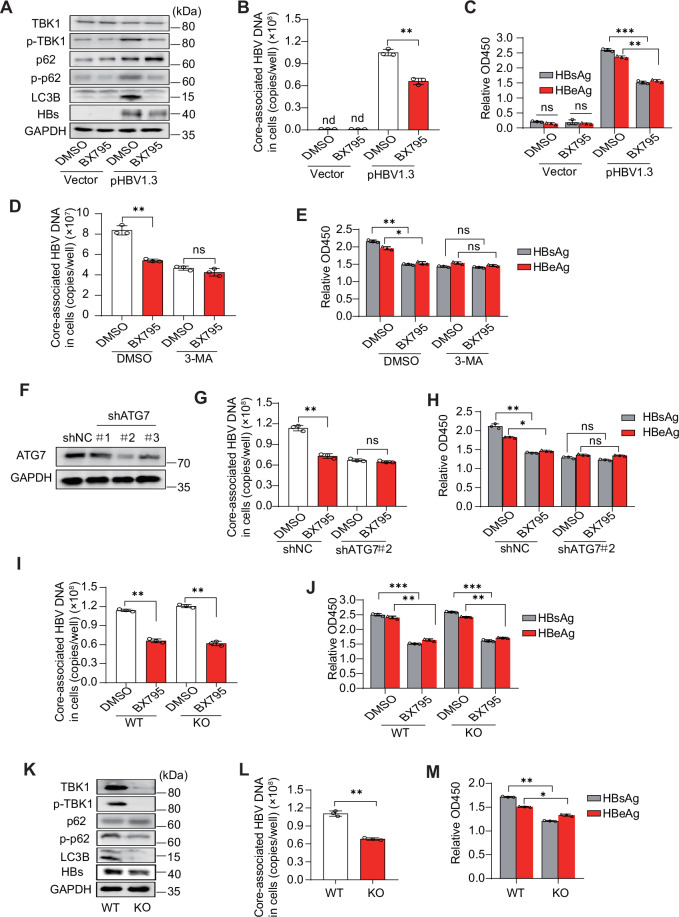
HBV replication relies on the activity of TBK1 to regulate autophagy. **A**–**C** Huh7 cells were transfected with an empty vector or pHBV1.3 for 12 h, then treated with or without BX795 (1 μM) for 36 h. Levels of indicated protein (**A**) core-associated HBV DNA (**B**) supernatant HBsAg, and HBeAg (**C**) were determined. **D**, **E** Huh7 cells were transfected with pHBV1.3 for 12 h, then treated with 3-MA (2.5 mM) and combined with or without BX795 for 36 h. Levels of core-associated HBV DNA (**D**) supernatant HBsAg, and HBeAg (**E**) were determined. **F** Huh7 cells were transfected with shRNA plasmid targeting noncoding RNA (shNC) and three shATG7 plasmids for 36 h. Immunoblot analysis of ATG7 proteins. **G**, **H** Huh7 cells were transfected with pHBV1.3 for 12 h, treated with or without BX795, and transfected with shATG7 or shNC for 36 h. Levels of core-associated HBV DNA (**G**), supernatant HBsAg, and HBeAg (**H**) were determined. **I**, **J** WT or *IFNAR1* knockout Huh7 cells were transfected with pHBV1.3 for 12 h, then treated with or without BX795. Levels of core-associated HBV DNA (**I**) supernatant HBsAg, and HBeAg (**J**) were determined. **K**–**M** *TBK1* knockout or WT Huh7 cells were transfected with pHBV1.3 for 48 h. Levels of indicated proteins (**K**) core-associated HBV DNA (**L**) supernatant HBsAg, and HBeAg (**M**) were determined. All experiments were repeated at least three times with consistent results. Bar graphs show the means ± SD (*n* = 3 biological replicates). ^*^P < 0.05; ^**^P < 0.01; ^***^P < 0.001; nd not detected; ns not significant; using Student’s *t*-test.

Autophagy is associated with HBV replication [46]. To determine whether TBK1 activity affects HBV replication, Huh7 cells were transfected with the pHBV1.3 plasmid and treated with BX795. Immunoblotting revealed that BX795 inhibited TBK1, p62 phosphorylation, and LC3-I/II conversion (Fig. 6A). BX795 also inhibited core-associated HBV DNA and secretion into the supernatant of HBsAg and HBeAg (Fig. 6B, C). 3-MA has been widely used as an autophagy inhibitor based on its inhibitory effect on class III PI3K activity [47]. Next, cells were transfected with pHBV1.3 and combined to be treated with BX795 and 3-MA. 3-MA treatment diminished the suppression of BX795 on HBV DNA and secreted HBsAg and HBeAg (Fig. 6D, E). As 3-MA is not a specific autophagy inhibitor, we designed three shRNAs (shRNA-ATG7#1, #2, and #3) to knock down autophagy-related 7 (*ATG7*) to block the autophagy pathway [48]. Then, we tested their efficiency (Fig. 6F). ShRNA-ATG7#2 was selected for the experiments. Consistently, *ATG7* knockdown diminished the suppression of BX795 on core-associated HBV DNA and secreted HBsAg and HBeAg (Fig. 6G, H). BX795 targeting TBK1 may hinder autophagy initiation and repress type I interferons. To exclude the effect of type I interferon on HBV replication, we treated *INFAR1* knockout Huh7 cells with BX795. The *INFAR1* knockout did not affect the effect of BX795 on HBV replication (Fig. 6I, J). These data suggest that HBV replication mainly relies on the TBK1-mediated autophagy pathway rather than the interferon pathway. To further confirm the effect of TBK1 activity on HBV replication, *TBK1* knockout and WT Huh7 cells were transfected with pHBV1.3 plasmid. The results showed that *TBK1* knockout decreased the initiation activity of autophagy and HBV replication (Fig. 6K–M). In conclusion, these findings suggest that HBV replication relies on TBK1 activity to regulate autophagy.

### HBsAg modulates innate immunity and autophagic flux in HBs-Tg mice

**Fig. 7:**
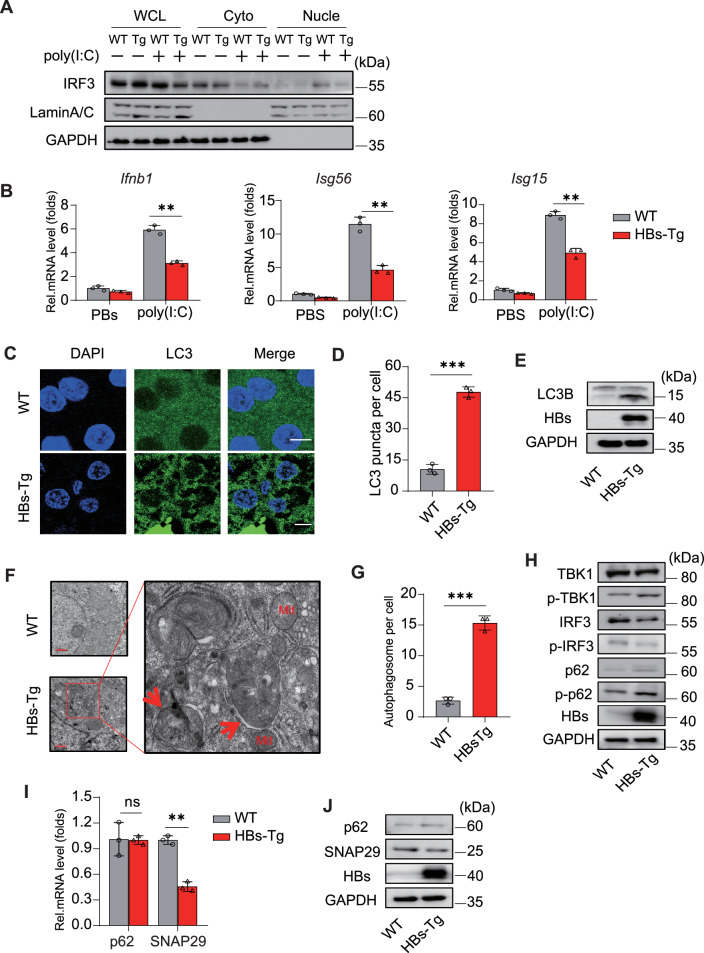
Assessment of innate immunity and autophagic flux in vivo. **A**, **B** PBS or poly (I:C) was injected at 5 mg/kg body weight via the tail vein in 9-week-old WT and HBs-transgenic mice. After 24 h, **A** immunoblot analysis of IRF3 in cytoplasmic (Cyto) and nuclear (Nucle) fractions. GAPDH served as a cytoplasmic control. LaminA/C served as a nuclear protein control. **B** Gene expression of interferon (*Ifnb1*) and ISGs (*Isg56*, *Isg15*) was determined by qRT-PCR. **C** Immunofluorescence staining analyses of LC3 in mouse liver tissue. **D** LC3-puncta were assessed by Image J. Scale bar: 10 μm. **E** Immunoblot analysis of LC3B in mouse liver tissue. **F** Observation of autophagosomes in WT and HBs-Tg mouse liver tissue by TEM, and the red arrow indicates autophagosomes with double membranes. Scale bar: 1 μm. **G** Quantification of the number of autophagic vacuoles observed by TEM. **H** Immunoblot analysis of (p)-TBK1, IRF3, and p62 in liver tissue from HBs-Tg and WT mice. **I**, **J** qRT-PCR (**I**) and immunoblot (**J**) analysis of p62 and SNAP29 mRNA and protein levels in mouse liver tissue. All experiments were repeated at least three times with consistent results. Bar graphs show the means ± SD (*n* = 3 biological replicates). ^**^P < 0.01; ^***^P < 0.001; ns not significant; using Student’s *t*-test.

HBsAg transgenic mice (HBs-Tg) contain a sequence that encodes the HBV large envelope polypeptide inserted downstream of the albumin promoter. Therefore, the peptide can be expressed in hepatocytes. To study the modulation of innate immune response by HBsAg in vivo, 9-week-old mice (*n* = 3) were injected in the tail vein with 5 mg/kg body weight poly (I:C), and PBS was injected in the control group. After 24 h, liver tissue was collected for subcellular fraction extraction and qRT-PCR. Compared to WT, poly (I:C)-induced nuclear translocation of IRF3 and mRNA levels of *Ifnb1*, *Isg15*, and *Isg56* were significantly inhibited in HBs-Tg mice (Fig. 7A, B). Next, we examined the autophagic flux in liver tissues. Immunofluorescence showed that LC3-positive puncta were increased in transgenic mice (Fig. 7C, D). Additionally, immunoblot showed increased LC3B-I/II conversion, and TEM showed increased double-membrane autophagosomes, which are present in the early stages of autophagy (Fig. 7E–G). TBK1 phosphorylates IRF3 to induce IFNβ or p62 to promote autophagy [26, 27]. Consistent with the findings in the cellular, immunoblotting of transgenic mouse livers showed increased phosphorylation of TBK1 and p62 proteins, whereas the phosphorylation level of IRF3 protein was decreased (Fig. 7I). These data suggest that HBsAg prevents IRF3-mediated IFNβ signaling but induces p62-mediated autophagy in the mouse liver. Subsequently, we also explored SNAP29 expression in liver tissues. SNAP29 but not p62 expression was downregulated in HBs-Tg mice (Fig. 7J, K). These findings suggest HBsAg attenuates innate immunity but induces early autophagy in vivo.

### Comparison of innate immunity and autophagic flux between chronic HBV and non-HBV patients

**Fig. 8:**
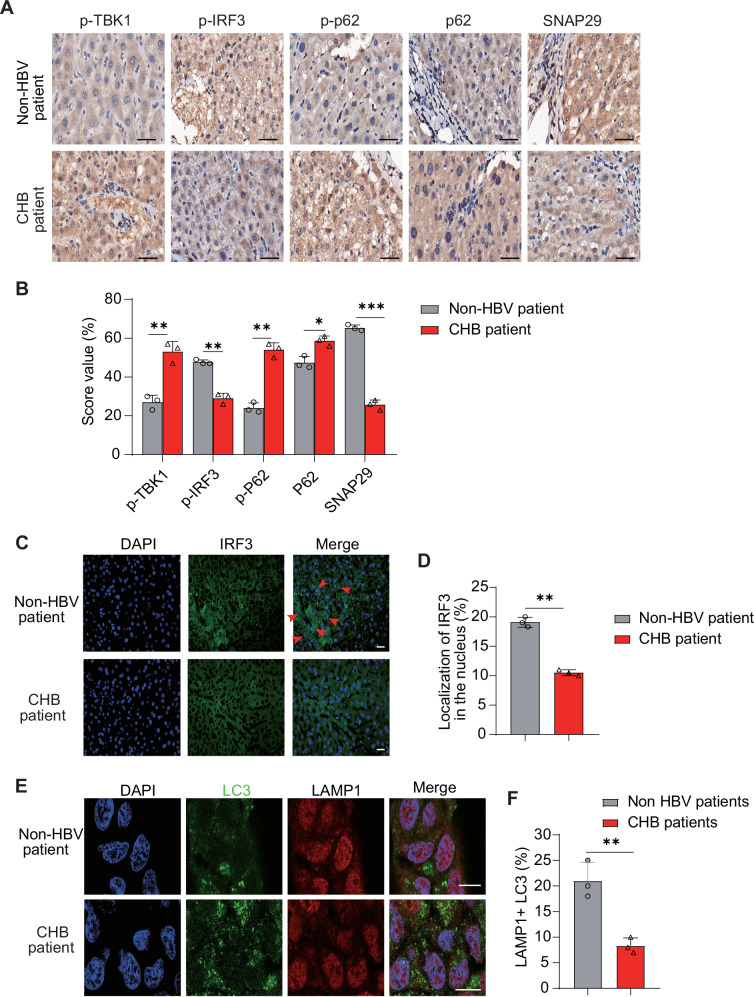
Changes in innate immunity and autophagic flux in liver tissue of CHB patients. **A** Immunohistochemical staining of p-TBK1, p-IRF3, p-p62, p62, and SNAP29 in bioptic liver tissue from chronic HBV (CHB) or non-HBV patients. Bar: 50 μm. **B** Assessment of p-TBK1, p-IRF3, p-p62, p62, and SNAP29 protein staining by score values using ImageJ. **C**–**F** Immunofluorescence staining analysis of IRF3 (**C**) or LC3 and LAMP1 (**E**) in liver tissue from CHB and non-HBV patients. Assessment of the IRF3 nuclear localization (**D**) and co-localization between LC3 and LAMP1 (**F**) by ImageJ. Red arrows indicated some nuclear localization of IRF3. Scale bar: 30 μm. All experiments were repeated at least three times with consistent results. Bar graphs show the means ± SD (*n* = 10 biological replicates). ^*^P < 0.05; ^**^P < 0.01; ^***^P < 0.001; using Student’s *t*-test.

To investigate whether HBV infection leads to changes in innate immunity and autophagic flux in the human liver, we recruited chronic HBV patients (*n* = 10) and non-HBV patients (*n* = 10) and collected their liver tissues. Immunohistochemistry revealed an increased phosphorylation of TBK1 and p62 proteins in the livers of HBV-infected patients, whereas the phosphorylation of IRF3 was decreased (Fig. 8A). In addition, there was a moderately increased expression of p62 and a decreased expression of SNAP29 in CHB patient liver tissues (Fig. 8A). The distribution of IRF3 and LC3 in liver tissues showed a decrease in IRF3 nucleation and in LC3 positive puncta for LAMP1 after HBV infection (Fig. 8C–F). These data suggest that innate immunity is downregulated and autophagosomes are accumulated in the liver tissues of CHB patients.

## Discussion

HBV infection of hepatocytes rarely triggers innate immunity, and cytokines are maintained at low levels [49, 50]. On the other hand, HBV infection activates autophagy and utilizes the autophagic pathway to aid viral replication [22, 48]. However, the precise molecular mechanisms remain unclear. In this study, we demonstrated suppression of the innate immune response and accumulation of autophagosomes by HBV and HBs in vivo and in vitro. HBsAg disrupts the TBK1–IRF3 interaction but enhances TBK1 dimerization, which is essential for TBK1 and p62-mediated autophagy. Moreover, HBs inhibit SNAP29 promoter activity, thereby suppressing autophagic degradation.

HBV DNA and HBsAg have been detected in macrophages of CHB patients [51]. HBs expression in THP-1 cells significantly inhibited type I interference and IRF3 phosphorylation, but TBK1 phosphorylation was upregulated. TBK1 was first discovered to play a role in the IFN signaling pathway [52]. TBK1’s activity is strictly regulated, including dimerization, ubiquitination, phosphorylation, and the formation of functional complexes [23, 25, 53, 54]. The KD is essential for its kinase activity, which is required to interact with IRF3 [55]. Our findings suggest that the KD is necessary for the interaction between TBK1 and HBsAg, which competitively impairs IRF3 binding. All three types of HBsAg interact with TBK1, and they contain common S sequences. Therefore, the S domain could competitively prevent the TBK1–IRF3 complex from downregulating type I interferon signaling.

On the other hand, TBK1 in autophagy and its phosphorylation of autophagy receptors, such as p62, NDP52, TAX1BP1, and OPTN, have gained significant interest [27, 56, 57]. A study reported that TRIM23 promotes TBK1 dimerization during viral infection and mediates autophagy through the TBK1-p62 axis [27]. TBK1^EE^ and TBK1^HIF^ mutations lead to defective dimerization [23]. As anticipated, HBs boost TBK1-WT dimerization; however, TBK1^EE^ and TBK1^HIF^ mutants are not facilitated. Enhanced TBK1 dimerization promotes p62 phosphorylation at serine 403, which induces autophagy [27]. These results imply that the TBK1-p62 axis may play a role in HBV infection.

Yuan et al. found that HBV small surface antigen is an essential protein for the induction of autophagy and is sufficient to trigger UPR signaling to induce autophagy [42]. Treatment with BX795, but not 4-PBA (an ER stress inhibitor), abolished the HBsAg-induced early phase of autophagy. This revealed that HBs initiate autophagy through an additional pathway and sheds light on the modulation between innate immunity and autophagy in HBV infection. Induction of early stages of autophagy can facilitate HBV replication. Sir et al. believed that the steps at or after the onset of autophagy were essential for HBV DNA replication and had little effect on HBV core RNA (pgRNA) [48]. HBs or HBV overexpression was found to promote TBK1, p62 phosphorylation, and LC3 conversion. Still, this phenomenon disappeared when TBK1 was knocked out and reappeared when WT, but not TBK1^EE^ and TBK1^HIF^ mutations, were restored in the *TBK1* knockout Huh7 cell line. In this study, we found that HBs promotes TBK1 dimerization and activates TBK1, which subsequently phosphorylates p62, initiating the early stages of autophagy, a process that supports HBV replication; however, this phenomenon disappeared when TBK1 was knocked out. This confirms that HBV-enhanced TBK1 dimerization induces p62-mediated autophagy to promote viral replication.

Some viruses have evolved strategies to benefit from autophagy to evade the innate immune response and establish a successful infection. For example, the hepatitis C virus suppresses innate immunity via autophagic degradation of TRAF6 [58]. SARS-CoV-2-induced mitochondria-mediated degradation of MAVS evades innate immunity [59]. A recent study showed that HBx inhibits the cGAS-STING pathway through autophagic degradation of cGAS [60]. The current research indicates that HBV inhibits autophagic degradation to induce incomplete autophagy [31]. Lu et al. reported that increased overexpression of SNAP29 promotes autophagic degradation of the HBV, and conversely, silencing of its expression is beneficial for HBV replication [32]. Our findings suggest that HBsAg hinders the transcription of SNAP29, suggesting that HBV employs a unique mechanism to evade autophagic degradation. Unlike other viruses, it may be an inefficient strategy for HBV to evade the innate immune response through autophagic degradation because HBV viral particles and proteins replicating and assembling in autophagosomes are degraded by fusion with lysosomes [32, 33, 46].

In conclusion, our findings indicate that TBK1 functions as a mediator to regulate innate immunity and autophagy during HBV infection, which may contribute to eliminating persistent HBV infection.

## Supplementary information

Supplementary Data

## Source data

Source Data

## Data Availability

All other data from the authors are available upon request. Source data are provided in this paper.
