# Supplementary material for: Hepatitis B surface antigen hijacks TANK-binding kinase 1 to suppress type I interferon and induce early autophagy
Source: Cell Death Dis. 2025 Apr 15;16(1):304. doi: 10.1038/s41419-025-07605-0 (PMC12000394; doi:10.1038/s41419-025-07605-0)
Supplement: Supplementary file 1 — Supplementary Data [file 41419_2025_7605_MOESM1_ESM.docx]

**Supplementary data:**

**Hepatitis B surface antigen hijacks TANK-binding kinase 1 to suppress type I interferon and induce early autophagy**

**
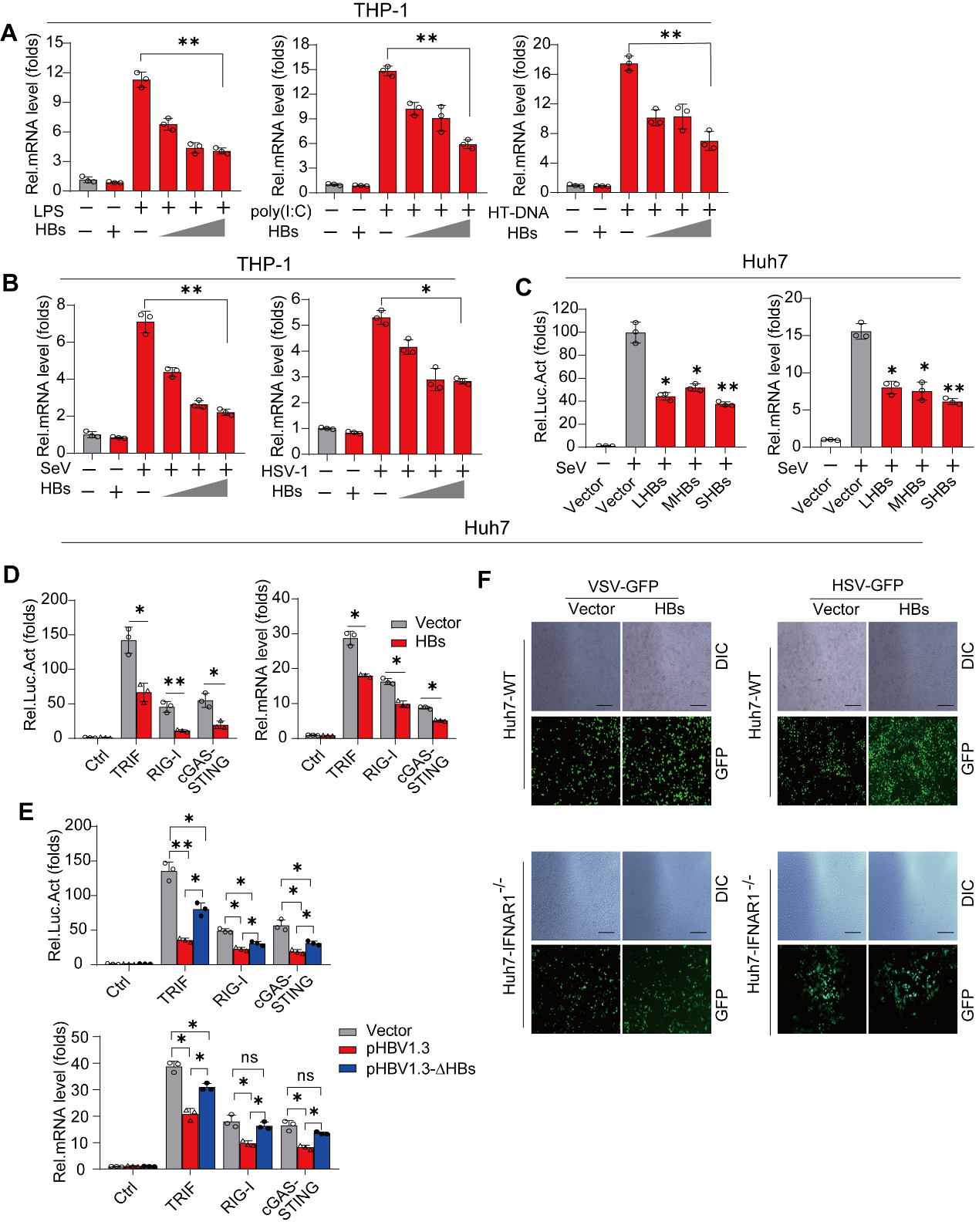
**

**Figure S1**. HBsAg negatively regulates the IFNβ signaling pathway.

(**A**) THP-1 cells were transfected with different doses (0, 1, 0, 0.2, 0.5, 1 μg) of HBs expression plasmids, and the total amount of plasmids was adjusted to 1 μg with an empty vector. After 24 h, cells were stimulated with LPS, poly(I: C), or HT-DNA for 8 h. The mRNA levels of *IFNB1* were detected by qPCR. (**B**) THP-1 cells were transfected with different doses (0, 1, 0, 0.2, 0.5, 1 μg) of HBs expression plasmids, and the total amount of plasmids was adjusted to 1 μg with an empty vector. After 24 h, the cells were infected with SeV (MOI of 1) or HSV-1 (MOI of 1) for 8 h. The mRNA levels of *IFNB1* were detected by qPCR. SeV, Sendai virus; HSV-1, Herpes simplex virus. (**C**) Huh7 cells were transfected with a plasmid encoding LHBs, MHBs, SHBs, or an empty vector, along with or without an *IFNB1* reporter and a Renilla-TK reporter, and then infected with SeV. The luciferase activity and mRNA levels of *IFNB1* were detected. (**D-E**) Huh7 cells were transfected with expression plasmids for TRIF, RIG-I, or cGAS+STING, vector or HBs (**D**), and pHBV1.3 or pHBV1.3-ΔHBs (**E**) for 24 h, and then together with or without an *IFNB1* reporter and a Renilla-TK reporter. Luciferase activity and mRNA levels of *IFNB1* were detected. (**F**) WT or *IFNAR1* knockout Huh7 cells were transfected with the vector or HBs expression plasmids for 24 h and then infected with VSV-GFP (MOI of 0.1) or HSV-GFP (MOI of 1) for 24 h. The VSV-GFP or HSV-GFP replication was visualized by fluorescence microscopy for GFP expression. Scale bar: 100 μm. VSV, Vesicular stomatitis virus. All experiments were repeated at least three times with consistent results. Bar graphs show the means ± SD (*n* = 3 biological replicates). *P < 0.05; **P < 0.01; ns, not significant; using Student’s t-test.


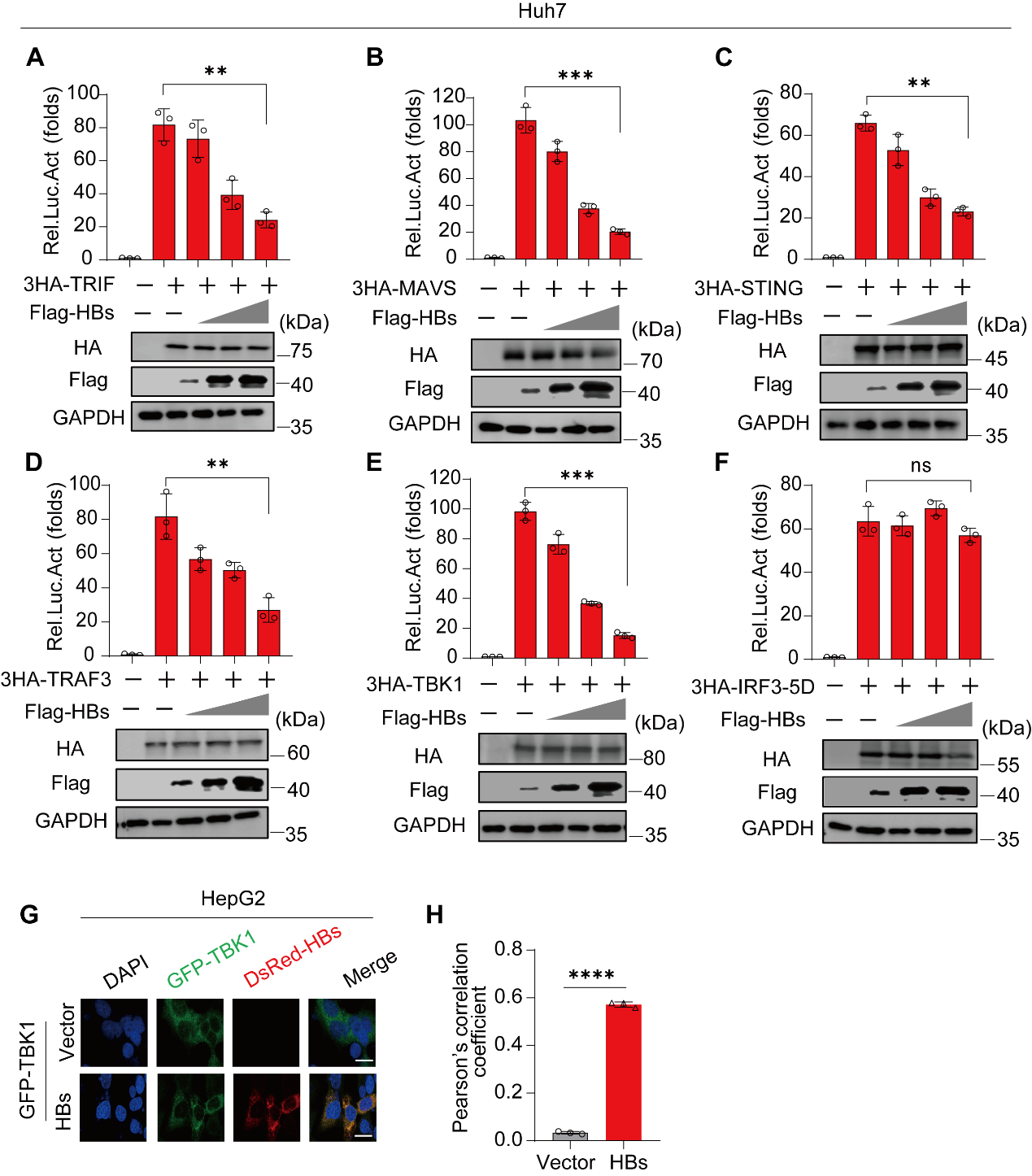


**Figure S2**. HBsAg suppresses the IFNβ promoter via TBK1.

(**A-F**) Huh7 cells were transfected with an IFNβ reporter, a Renilla-TK reporter, and a plasmid expressing HA-tagged TRIF (**A**), MAVS (**B**), STING (**C**), TRAF3 (**D**), TBK1 (**E**), or IRF3-5D (**F**), along with increasing amounts of a plasmid encoding Flag-HBs. Luciferase activity of IFNβ was detected. Immunoblot analysis of the expression levels of indicated proteins. (**G**) HepG2 cells were transfected with a plasmid encoding GFP-TBK1 and DsRed-HBs or vector for 48 h. TBK1 and HBs co-localization was observed by confocal microscopy. Scale bar: 20 μm (**H**) Statistical co-localization analysis between HBsAg and TBK1. All experiments were repeated at least three times with consistent results. Bar graphs show the means ± SD (*n* = 3 biological replicates). **P < 0.01; ***P < 0.001; ****P < 0.0001; ns, not significant; using Student’s t-test.


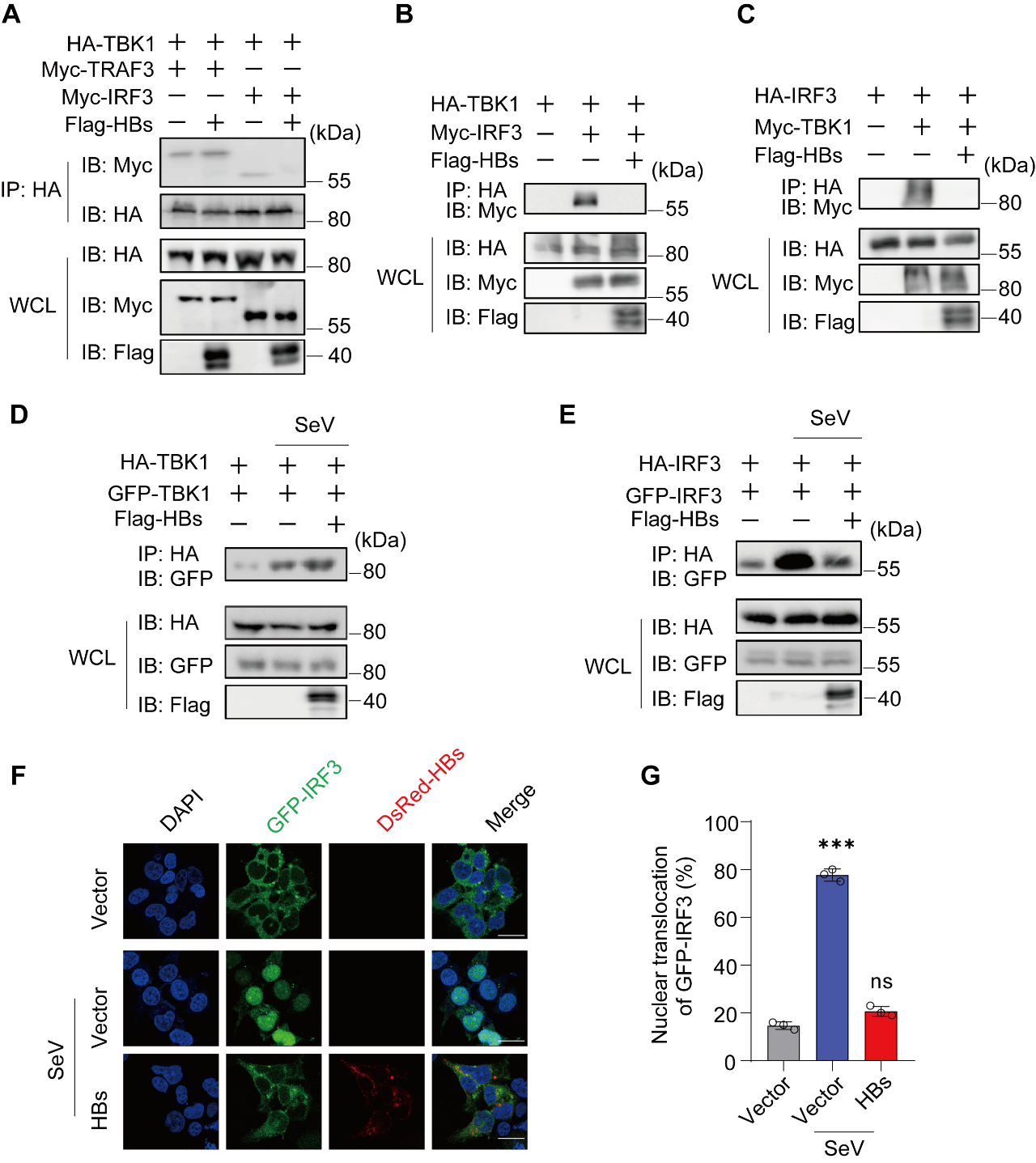


**Figure S3**. HBsAg disrupts TBK1-IRF3 interactions and inhibits IRF3 dimerization and nucleation.

(**A**) HepG2 cells were transfected with a plasmid encoding HA-TBK1 and Myc-TRAF3 or Myc-IRF3, along with or without Flag-HBs for 48 h. Cells were harvested to be analyzed by immunoprecipitation and immunoblot. (**B-C**) Immunoprecipitation and immunoblot in HepG2 cells transfected with plasmids encoding HA-TBK1 and Myc-IRF3 (**B**) or HA-IRF3 and Myc-TBK1 (**C**), with or without a plasmid encoding Flag-HBs for 48 h. (**D-E**) HepG2 cells were transfected with plasmids expressing HA-TBK1 and GFP-TBK1 (**D**) or HA-IRF3 and GFP-IRF3 (**E**) and with or without Flag-HBs and then stimulated with SeV for 9 h. Immunoprecipitation and immunoblot analysis of TBK1 and IRF3 dimerization. (**F**) HepG2 cells were transfected with plasmids expressing GFP-IRF3, DsRed-HBs, or a vector and then infected with SeV. IRF3 and HBs were identified as green and red signals and nuclear stained with DAPI (blue) by confocal microscopy. Scale bar: 20 μm. (**G**) Quantifying the percentages of IRF3 nuclear translocation by image J. All experiments were repeated at least three times with consistent results. Bar graphs show the means ± SD (*n* = 3 biological replicates). ***P < 0.001; ns, not significant; using Student’s t-test.


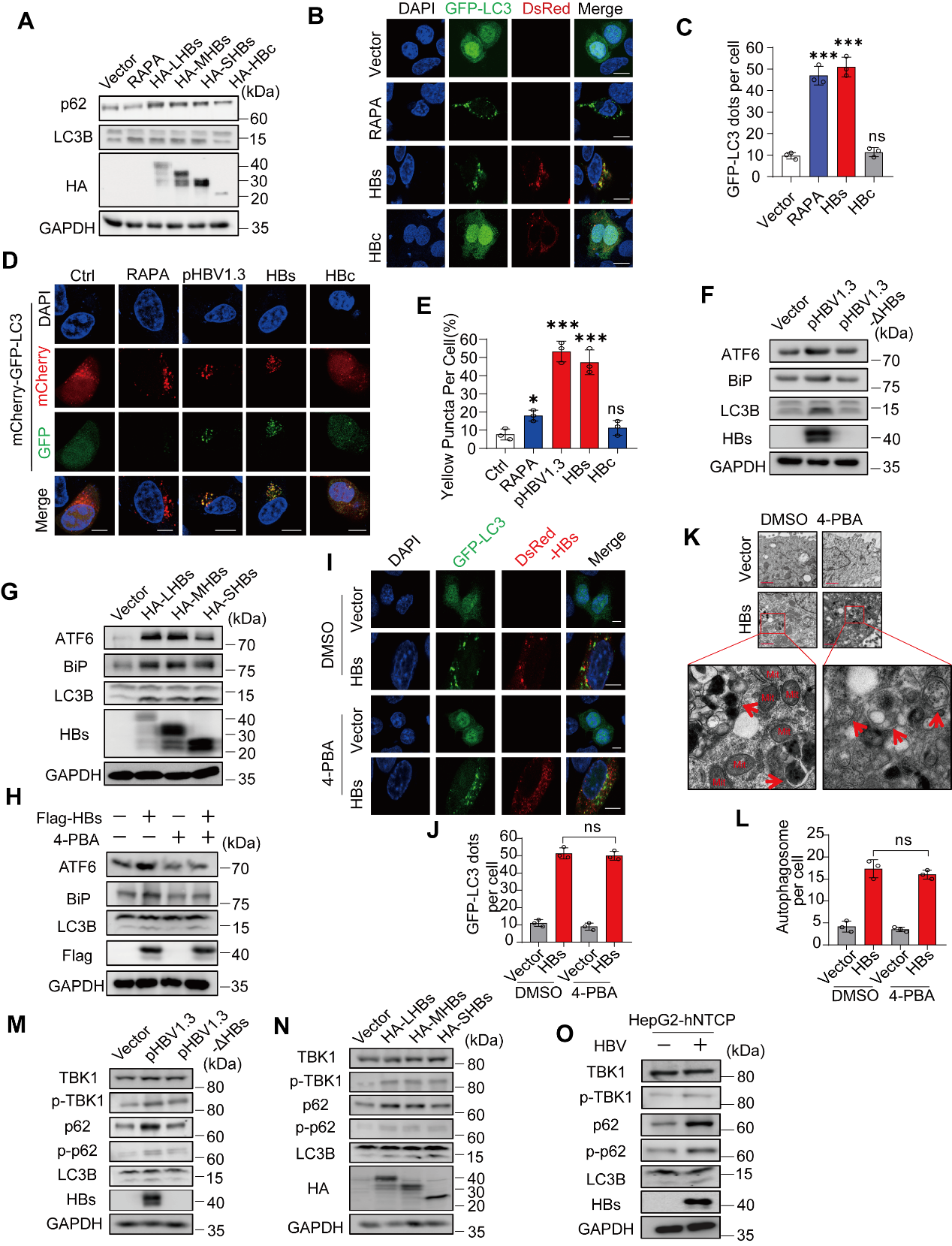


**Figure S4**. HBs induces autophagy initiation but impairs late stage of autophagy.

(**A**) Huh7 cells were transfected with a plasmid encoding HA-tagged LHBs, MHBs, SHBs, HBC, or an empty vector or stimulated with RAPA (2.5 μM). Immunoblot analysis of p62 and LC3B proteins. (**B**) Huh7 cells were transfected with a plasmid encoding DsRed-tagged HBs or HBc and GFP-LC3 or stimulated with RAPA (2.5 μM). GFP-LC3 puncta was examined by fluorescence microscopy. Scale bar: 10 μm. (**C**) The numbers of GFP-LC3 puncta per cell in (**B**) were quantified. (**D**) Huh7 cells expressing mCherry-GFP-LC3 plasmid were transfected with HBs, pHBV1.3, HBc, or empty vector plasmid for 48 h or treated with RAPA (2.5 μM), and then cells were imaged by confocal microscopy. Scale bar: 10 μm. (**E**) Quantifying the percentage of yellow signal positive for both mCherry puncta and GFP puncta per cell in (**D**). (**F**) Huh7 cells were transfected with pHBV1.3, pHBV1.3-ΔHBs plasmids, or an empty vector. Immunoblot analysis of indicated proteins. (**G**) Huh7 cells were transfected with a plasmid encoding HA-LHBs, HA-MHBs, HA-SHBs, or an empty vector. Immunoblot analysis of indicated proteins. (**H**) Huh7 cells were transfected with Flag-HBs plasmid and then treated with or without 4-phenylbutyric acid (4-PBA, 1 mM). Immunoblot analysis of indicated proteins. (**I**) HepG2 cells were transfected with a plasmid encoding GFP-LC3 and DsRed-HBs or vector and then treated with or without 4-PBA (1 mM). LC3 puncta was examined by fluorescence microscopy. Scale bar: 10 μm. (**J**) The GFP-LC3 positive puncta per cell in (**I**) were quantified. (**K**) Cells were treated as in (**I**), and then autophagic vacuoles were observed by transmission electron microscopy (TEM). Mit, mitochondria. Scale bar: 1 μm. (**L**) Quantitative analysis of the number of autophagic vacuoles observed by TEM. (**M**) Huh7 cells were transfected with pHBV1.3, pHBV1.3-ΔHBs plasmids, or an empty vector. Immunoblot analysis of indicated proteins. (**N**) Huh7 cells were transfected with a plasmid encoding HA-LHBs, MHBs, SHBs, or an empty vector. Immunoblot analysis of indicated proteins. (**O**) Immunoblot analysis of indicated proteins in HepG2-hNTCP cells infected with HBV. All experiments were repeated at least three times with consistent results. Bar graphs show the means ± SD (*n* = 3 biological replicates). ***P < 0.001; ns, not significant; using Student’s t-test.


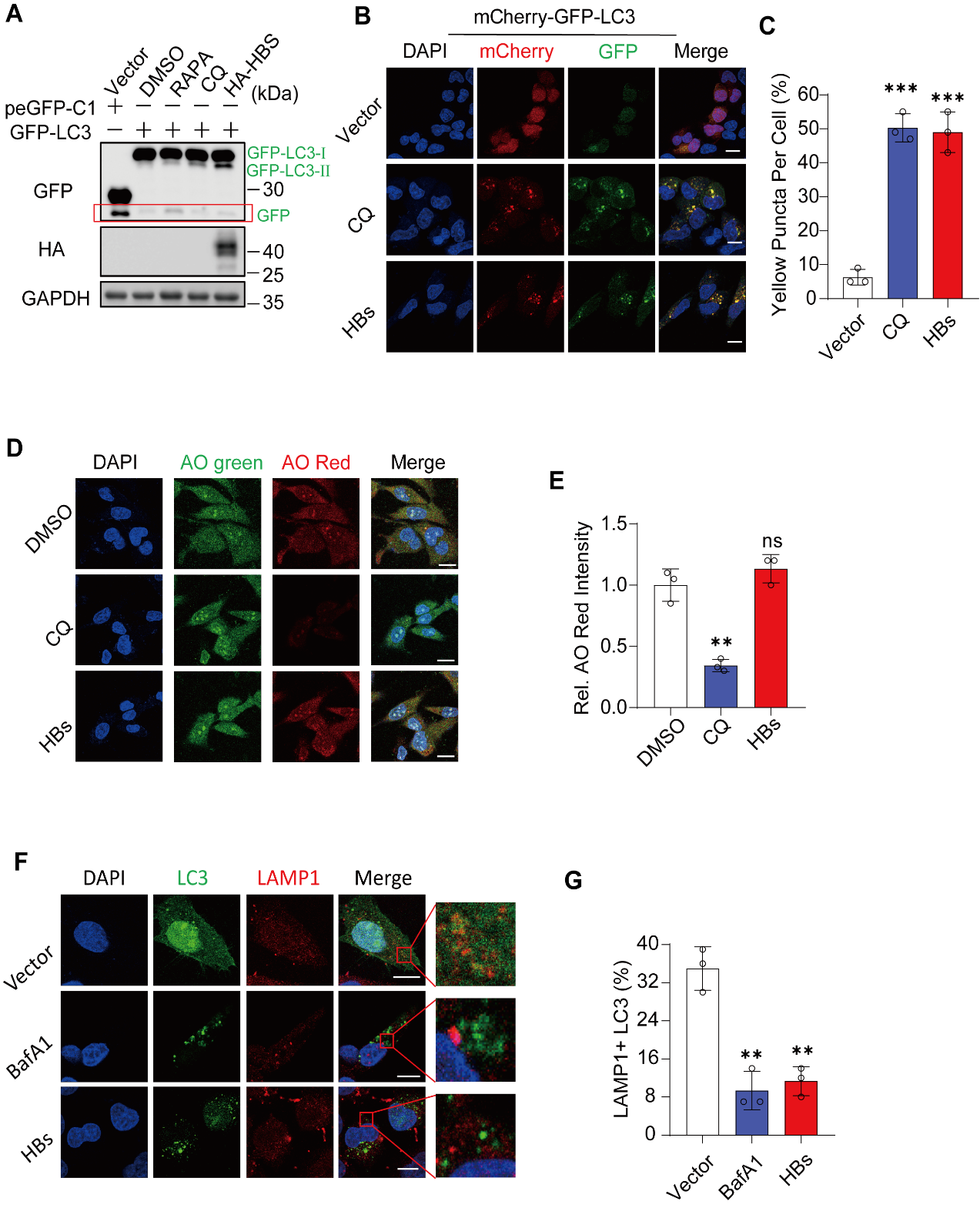


**Figure S5.** HBsAg induces incomplete autophagy in Huh7 cells.

(**A**) Huh7 cells were transfected with GFP-LC3 together with HA-HBs or treated with RAPA (2.5 μM) or CQ (10 μM). Cells were analyzed by immunoblot using anti-GFP antibodies. Note the production of GFP fragments. (**B**) Huh7 cells expressing mCherry-GFP-LC3 plasmid were transfected with HBs plasmid for 48 h or treated with CQ (10 μM) and then cells were imaged by confocal microscopy. Scale bar: 20 μm. (**C**) Quantifying the percentage of yellow puncta/cell positive for both mCherry and GFP in (**B**). (**D**) Huh7 cells were transfected with a plasmid encoding HBs or treated with CQ (10 μM). Cells were stained with AO for 15 minutes and then detected by confocal microscopy. Scale bar: 20 μm. (**E**) Statistical analysis of relative AO-red intensity. (**F**) Huh7 cells were transfected with HA-HBs plasmid or treated with BafA1 (100 nM). The co-localization of LC3 and LAMP1 was imaged. Scale bar: 10 μm. (**G**) Analysis of co-localization between LC3 and LAMP1 by imaged J. All experiments were repeated at least three times with consistent results. Bar graphs show the means ± SD (*n* = 3 biological replicates). **P < 0.01; ***P < 0.001; ns, not significant; using Student’s t-test.

**Table S1**. Characteristics of volunteers whose liver tissues were used in this study.

| Samples | Liver | |
| --- | --- | --- |
| Characteristic | Non-HBV Patients  (N=10) | HBV-infected Patients  (N=10) |
| Age (years) | 48.3±14.2 | 50.3±8.9 |
| Gender (male/female) | 7/3 | 9/1 |

**Table S2**. Primers were used for PCR in this study.

| Target | Application | Forward primer (5’ to 3’) | Reverse primer (5’ to 3’) |
| --- | --- | --- | --- |
| TBK1^S172A^ | S172A | gagaattagaagatgatgagcagtttgttGctctgtatggcacagaagaatatttgca | tgcaaatattcttctgtgccatacagagCaacaaactgctcatcatcttctaattctc |
| TBK1^EE^ | E355R | aattatttcttcaaatcaagaacttatctacCGagggcgacgcttagtcttaga | TctaagactaagcgtcgccctCGgtagataagttcttgatttgaagaaataatt |
|  | E448R | aaaggggatacgatggctgattCGattaattaaagatgattacaatgaaactgt | acagtttcattgtaatcatctttaattaatCGaatcagccatcgtatccccttt |
| TBK1^HIF^ | H459E | aaagatgattacaatgaaactgttGAGaaaaagacagaagttgtg | cacaacttctgtctttttCTCaacagtttcattgtaatcatcttt |
|  | I466E | aaaaagacagaagttgtgGAGacattggatttctgtatc | gatacagaaatccaatgtCTCcacaacttctgtcttttt |
|  | F470E | aagttgtgatcacattggatGAGtgtatcagaaacattgaaa | tttcaatgtttctgatacaCTCatccaatgtgatcacaactt |
| TBK1^K30R^ | K30R | caaatgtctttcgtggaagacataagaGaactggtgatttatttgctatcaaagtat | atactttgatagcaaataaatcaccagttCtcttatgtcttccacgaaagacatttg |
| TBK1^K401R^ | K401R | attaatatatgaaaaaatttccctccctaGagtacatccacgttatgatttagacgg | ccgtctaaatcataacgtggatgtactCtagggagggaaattttttcatatattaat |
| SNAP29 | Promoter | aaccCTCGAGtaccactgctggagctgcag | aattAAGCTTcaaccgcagcctactgtcca |
|  | Expression | attAAGCTTatgtcagcttaccctaaaagctacaatcc | aatTCTAGAgagttgtcgaacttttctttctgtgctt |
| TBK1-D1 | 1-309 | aatCTCGAGatgcagagcacttctaatcatctgtg | aatTCTAGAcattcggtgaagtatatcactagtttctgcaaa |
| TBK1-D2 | 310-386 | aatAAGCTTgtaattcatgttttttcgctacaacaaatgacag | aatTCTAGAtcaaggttcccggcttactacaaatataggg |
| TBK1-D3 | 310-729 | aatAAGCTTgtaattcatgttttttcgctacaacaaatgacag | aatGGATCCaaaagacagtcaacgttgcgaagg |
| HBs | DsRed-N1 | aatGGTACCatggggcagaatctttccaccag | aatACCGGTccaatgtatacccaaagacaaaagaaaattggtaacag |
| HBc | DsRed-N1 | aatGGTACCatggacatcgacccttataaag | aatACCGGTacacattgaggttcccgagattgaga |

Red stands for the mutation sites, Black and capital letters, restriction endonuclease enzyme cutting sites.

**Table S3**. Primers were used for qPCR analysis in this study.

| Target | Species | Forward primer (5’ to 3’) | Reverse primer (5’ to 3’) |
| --- | --- | --- | --- |
| *IFNB1* | Human | AAAGAAGCAGCAATTTTCAGC | CCTTGGCCTTCAGGTAATGCA |
| *Ifnb1* | Mouse | CAGCTCCAAGAAAGGACGAAC | GGCAGTGTAACTCTTCTGCAT |
| *Isg15* | Mouse | AAGGCTGTGGGCAAGG | TGGAGGAGTGGGTGTCG |
| *Isg56* | Mouse | CTGAGATGTCACTTCACATGGAA | GTGCATCCCCAATGGGTTCT |
| *SNAP29* | Human | TCATGTACGAGTCCGAGAAGG | CCCAAACACGCTCTTAATGCTAT |
| *Snap29* | Mouse | TTCGACGATGACGTGGAAGAG | GGTACTGCTGCCTGTCAATGG |
| *Rab7a* | Human | TACAAAGCCACAATAGGAGCTG | GCAGTCTGCACCTCTGTAGAAG |
| *LAPM1* | Human | CACGAGAAATGCAACACGTTAC | GGGTGCCACTAACACATCTGTAT |
| *VAPM8* | Human | TGTGCGGAACCTGCAAAGT | CTTCTGCGATGTCGTCTTGAA |
| *STX17* | Human | TCCTTTGACCAGATCCATGACT | CTTGAGGAATTTCAGGTAAGGCA |
| *PLEKHM1* | Human | CGGGCCATAAGATACGGAGGA | GCAATGGTCTGGACTCTTGGA |
| *LC3B* | Human | AAGGCGCTTACAGCTCAATG | CTGGGAGGCATAGACCATGT |
| *RILP* | Human | GAGCGGAATGAACTCAAAGCC | CCTCTGGTGTCCCTAACATCTTG |
| *VSV-P* | Virus | GTGACGGACGAATGTCTCATAA | TTTGACTCTCGCCTGATTGTAC |
| *HSV-P* | Virus | GCTCGAGTGCGAAAAAACGTTC | TGCGGTTGATAAACGCGCAGT |
| *HBV* | Virus | GGGAAACATAGAGGTTCCTTGA | GTTGCCCGTTTGTCCTCTAATTC |
| *GAPDH* | Human | AATGGGCAGCCGTTAGGAAA | GCGCCCAATACGACCAAATC |
| *Gapdh* | Mouse | AGGTCGGTGTGAACGGATTTG | TGTAGACCATGTAGTTGAGGTCA |
