# Supplementary material for: Hepatitis B surface antigen hijacks TANK-binding kinase 1 to suppress type I interferon and induce early autophagy
Source: Cell Death Dis. 2025 Apr 15;16(1):304. doi: 10.1038/s41419-025-07605-0 (PMC12000394; doi:10.1038/s41419-025-07605-0)

Figure1A

Anti-HA(M180-3)
Anti-Flag(M185-3L)
Anti-Myc(M192-3)
Anti-GFP(66002-1-Ig)

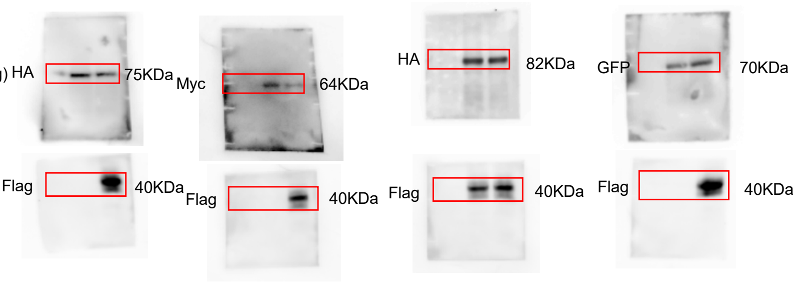

Figure1B

Anti-TBK1(A3458)
Anti-HBs(bs-1557G)
Anti-GAPDH (60004-1-Ig)

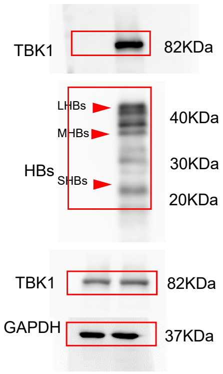

Figure 1F

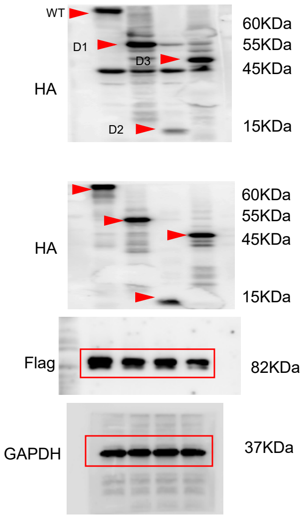

Figure 1H

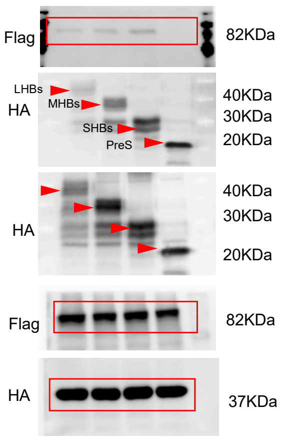

*

Figure2A

Anti-p-TBK1 (AP1418)
Anti-IRF3(A19717)
Anti-p-IRF3 (AP1412)
Anti-p62 (A19700)
Anti-p-p62 (YP1505)

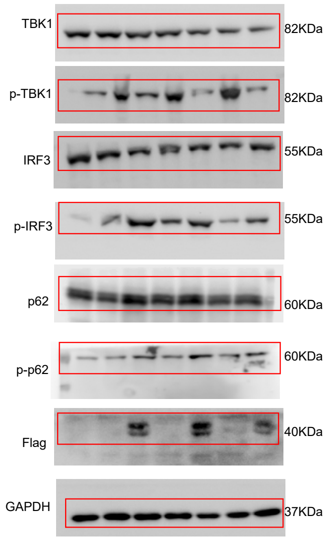

Figure2B

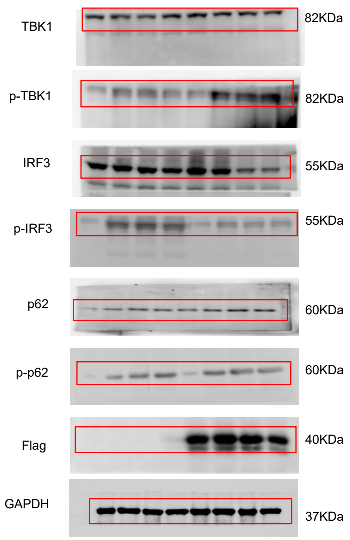

Figure2C

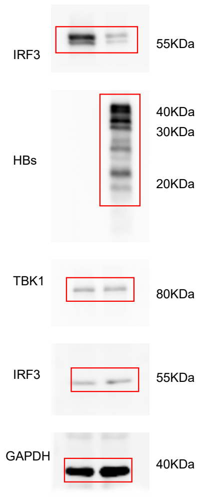

Figure2D

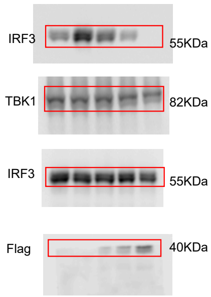

Figure2E

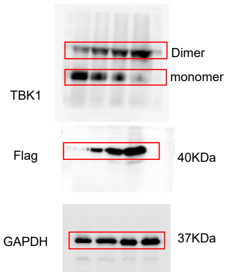

Figure2F

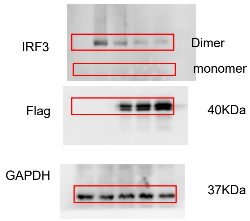

Figure2G

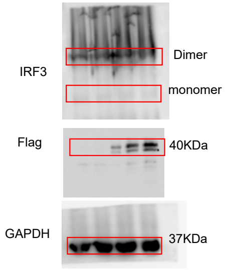

Figure2H

LaminA/C(10298-1-AP)

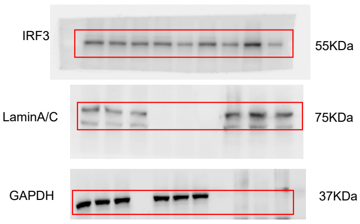

Figure2I

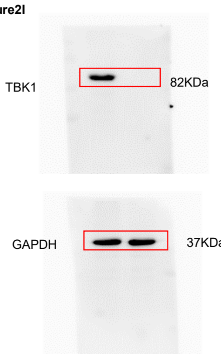

Figure2J

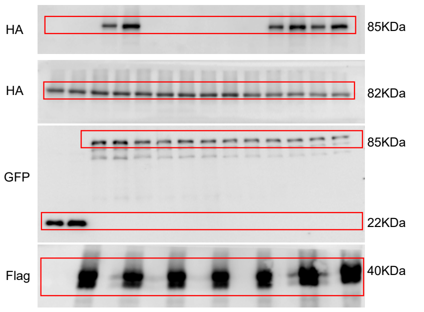

Figure3A

Anti-LC3B(A19665)

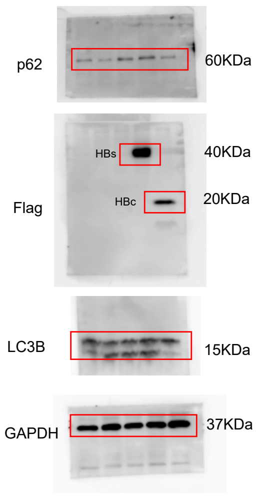

Figure3D

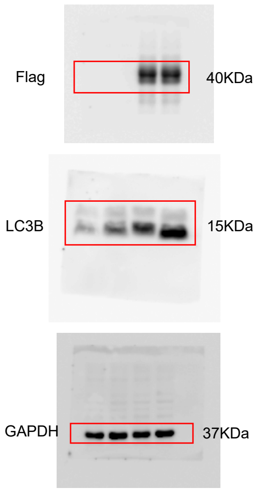

Figure3F

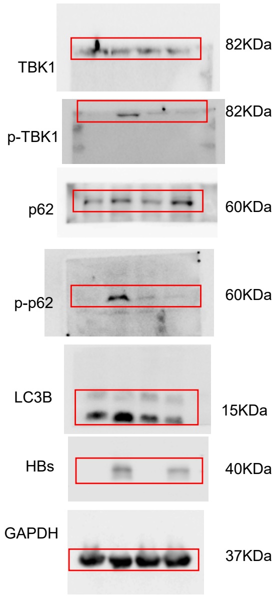

Figure3K

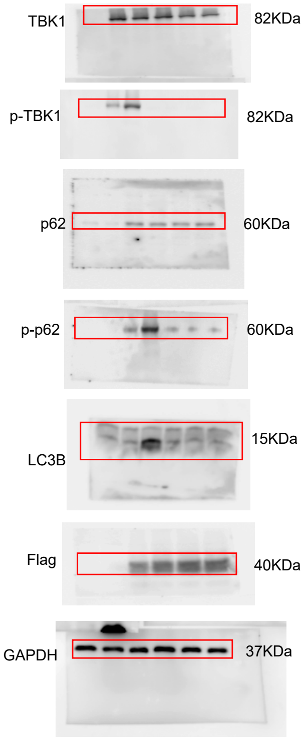

Figure4G

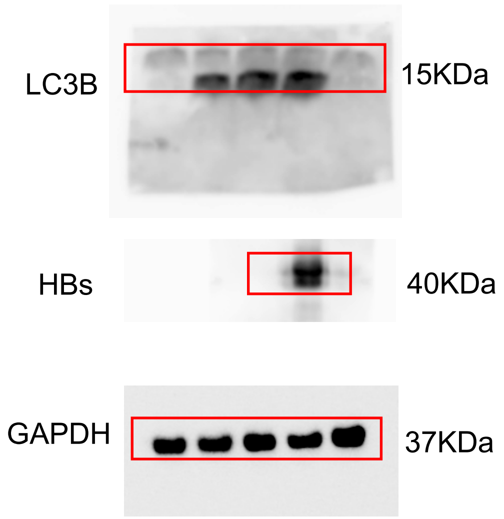

Figure5C

Anti-SNAP29(A4290)

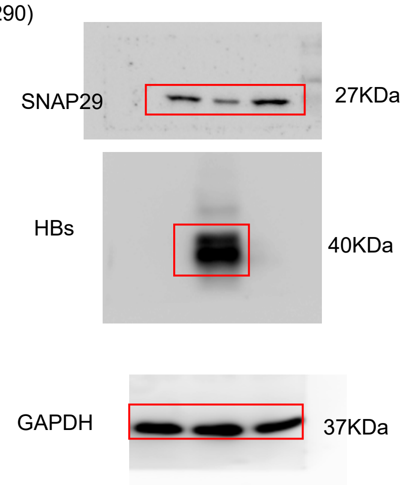

Figure5F

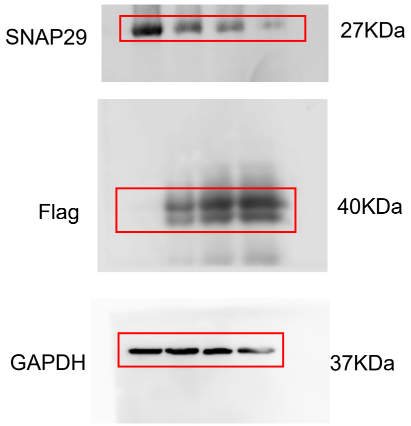

Figure6A

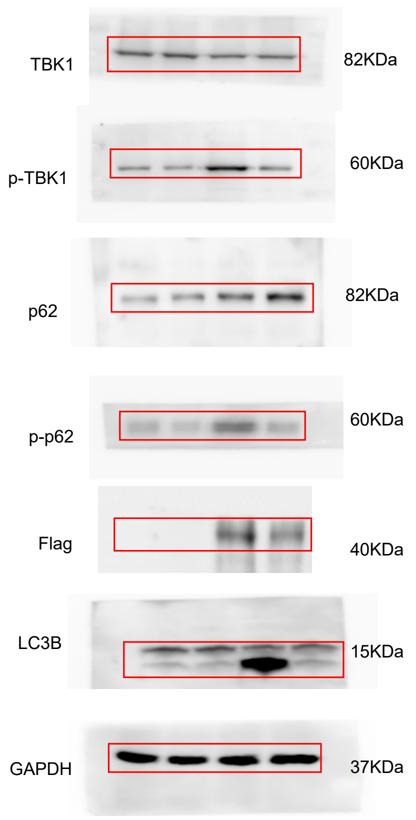

Figure6F

Anti-ATG7 (A19604)

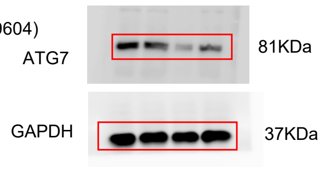

Figure6K

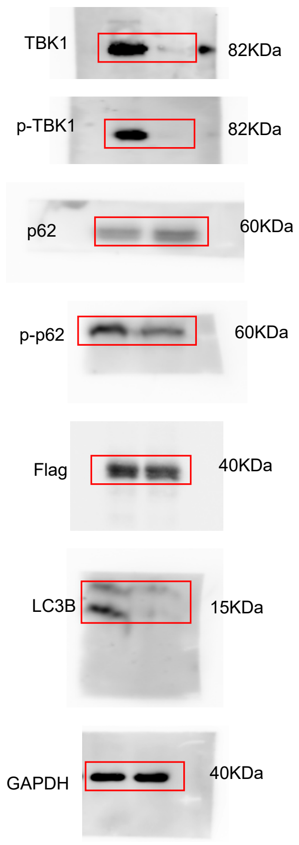

Figure7A

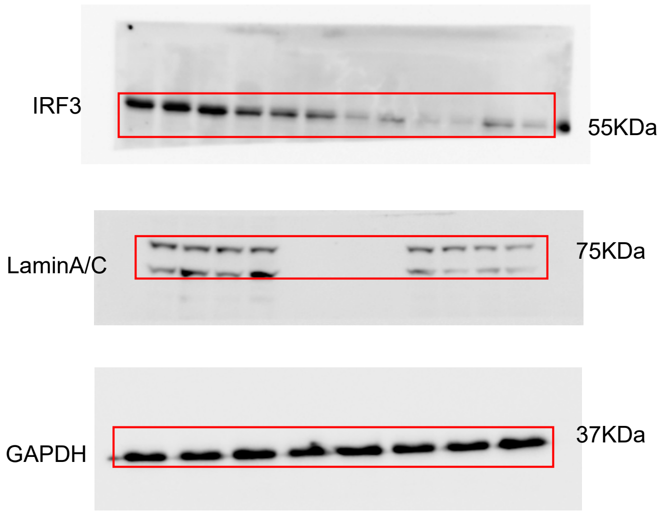

Figure7J

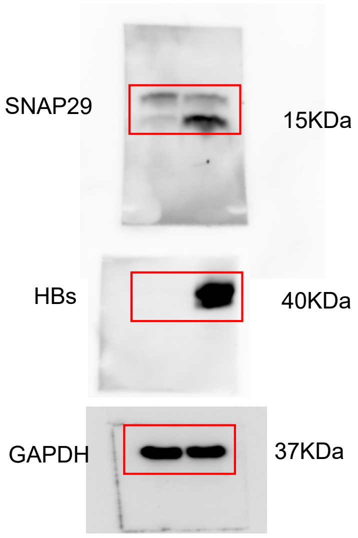

Figure7H

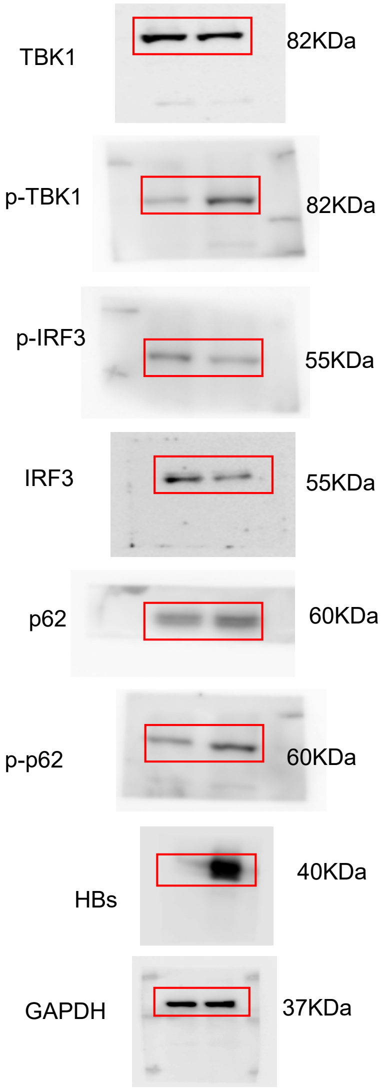

Figure7J

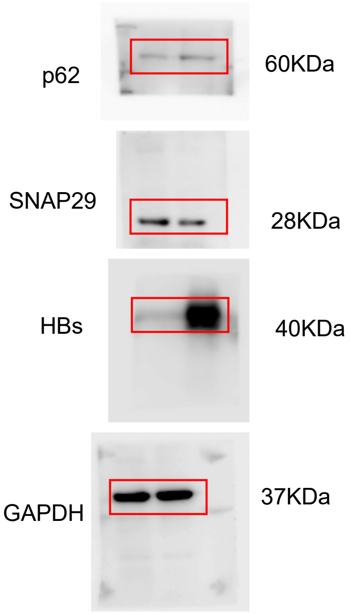

FigureS2A

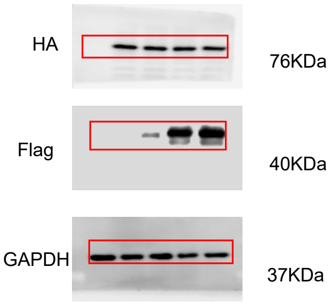

FigureS2B

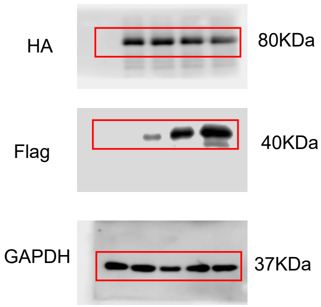

FigureS2C

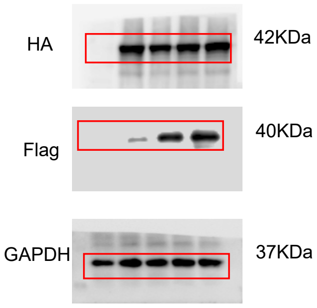

FigureS2D

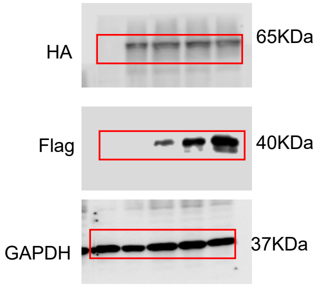

FigureS2E

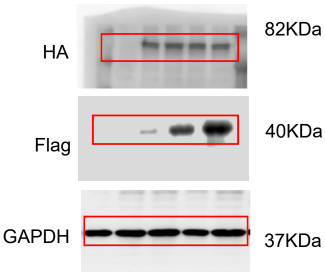

FigureS2F

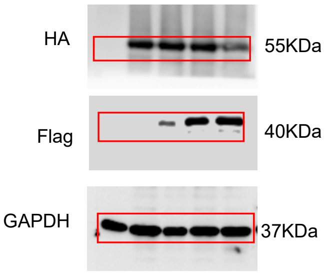

FigureS3A

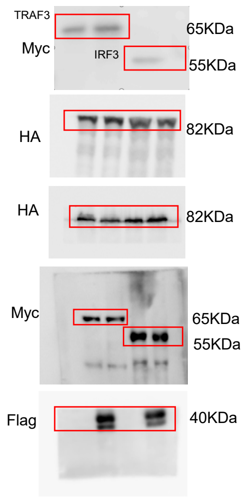

FigureS3B

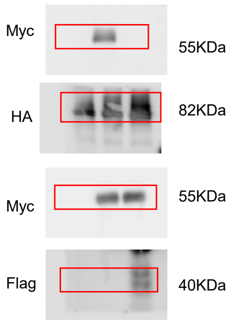

FigureS3C

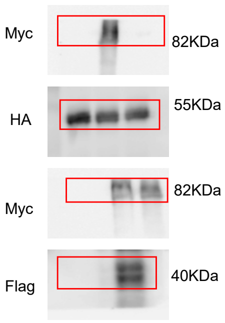

FigureS3D

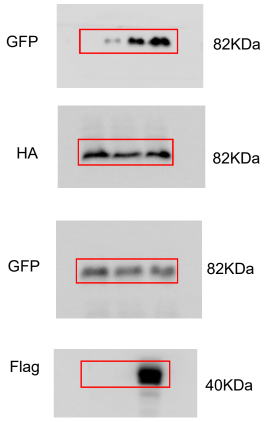

FigureS3E

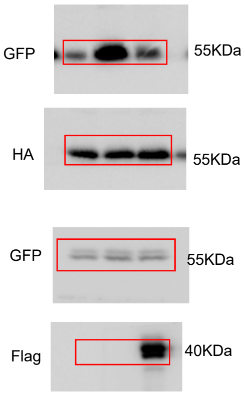

FigureS4A

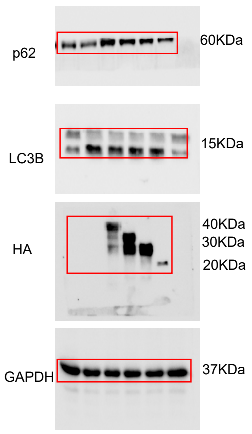

FigureS4F

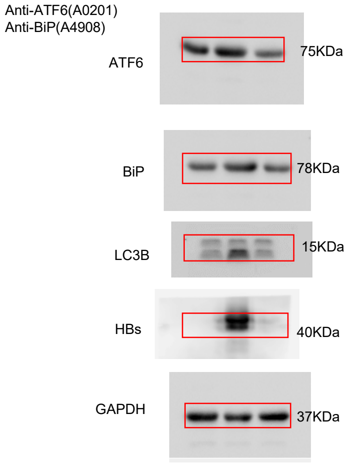

FigureS4G

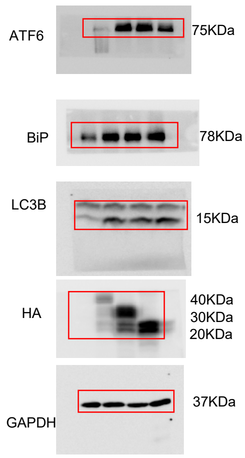

FigureS4H

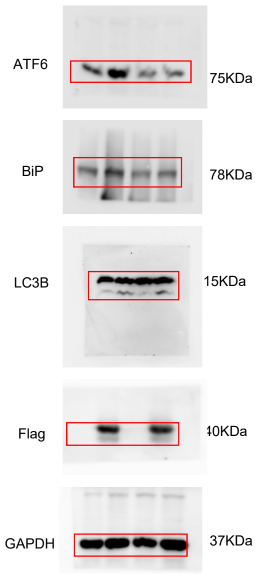

FigureS4M

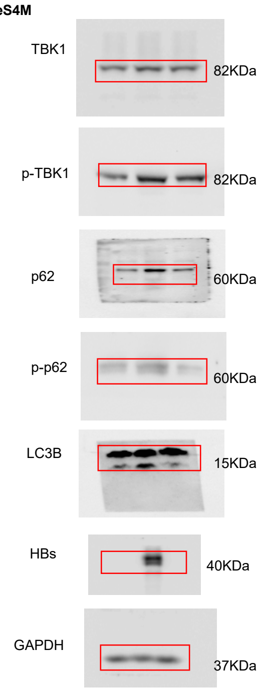

FigureS4N

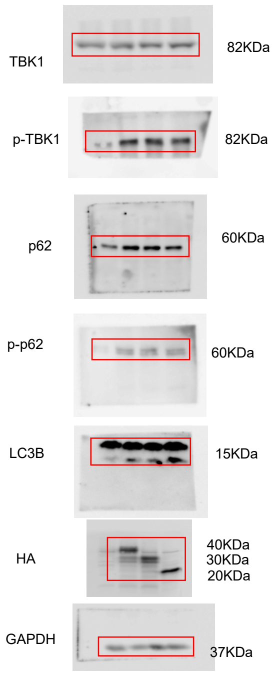

FigureS4O

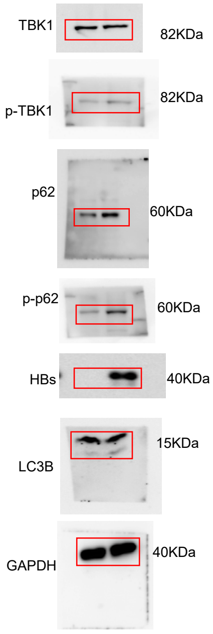

FigureS5A

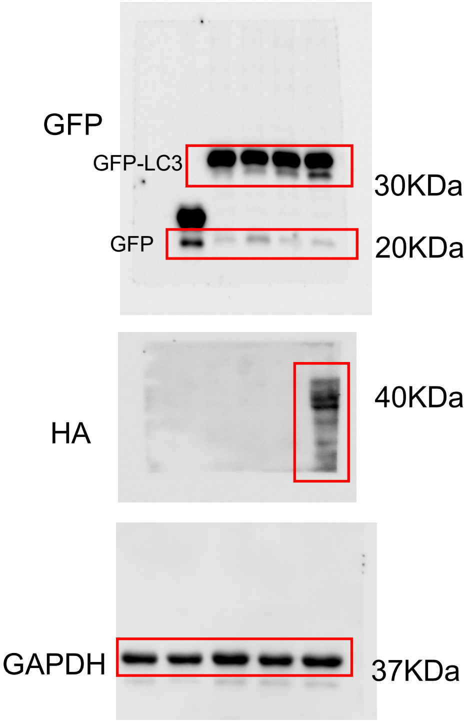

Supplement: Supplementary file 2 — Source Data [file 41419_2025_7605_MOESM2_ESM.pdf]
